# Design Rules for Open-Shell Molecular Wires: Insights from Correlated Many-Body Transport

**DOI:** 10.1021/acs.nanolett.6c02096

**Published:** 2026-07-27

**Authors:** Mehrdad Shiri, Leopoldo Mejía, Ignacio Franco, Thorsten Hansen, Kun Wang

**Affiliations:** † Department of Physics, 5452 University of Miami, Coral Gables, Florida 33146, United States; ‡ Departamento de Física y Astronomía, Facultad de Ciencias Exactas, 28087 Universidad Andres Bello, Santiago 837-0136, Chile; § Department of Chemistry, 6927 University of Rochester, Rochester, New York 14627, United States; ∥ Department of Physics and Astronomy, University of Rochester, Rochester, New York 14627, United States; ⊥ Institute of Optics, University of Rochester, Rochester, New York 14627, United States; # Department of Chemistry, University of Copenhagen, DK 2100 Copenhagen Ø, Denmark; 7 Department of Chemistry, University of Miami, Coral Gables, Florida 33146, United States

**Keywords:** Correlated transport, Open-shell molecular wire, Density matrix renormalization group, SSH-Hubbard model, Molecular electronics, Long-range charge transport

## Abstract

Open-shell π-conjugated systems have emerged as promising molecular wires that can sustain unusually high low-bias conductance over tens of nanometers. However, predictive design rules remain limited because near-degeneracy and spin polarization give rise to correlated electronic states that are not reliably captured by standard density functional theory and Landauer transport descriptions. Here, we establish physically transparent design rules for long-range, weakly length-dependent charge transport based on the interplay between bond-length alternation and electron–electron interactions. Using a fully correlated transport framework that combines density matrix renormalization group calculations with nonequilibrium Green’s function embedding, we show that weak dimerization maintains spatially extended edge states, while moderate on-site interaction stabilizes open-shell character without excessively separating transport-relevant resonances. This balance yields zero-bias transmission that is largely insensitive to molecular length, providing a route to connect chemically tunable structure to correlated transport beyond mean-field descriptions.

## Graphical abstract

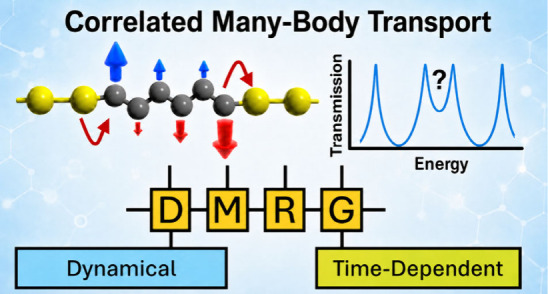

A predictive understanding of quantum effects in molecular-scale systems is central to the development of miniaturized electronic, spintronic, and quantum information technologies.^1–7^ In this context, theoretical tools based on single-particle frameworks for computing charge transport, such as density functional theory (DFT) in combination with the Landauer approximation, have been very successful in describing closed-shell, weakly correlated systems, enabling much of the progress in molecular electronics.^8–12^ However, an increasing number of molecular backbones with electronic features that are not well described by single-particle frameworks are emerging as new platforms for molecular electronics,^13–16^ challenging both the predictive power of state-of-the-art transport simulations and the physical intuition they have established.

In particular, open-shell π-conjugated systems have emerged as promising platforms for molecular wires that can sustain unusually high conductance over tens of nanometers at low bias, challenging the conventional expectation of exponential decay with length.^13^ In these systems, strong local electron–electron interactions and near-degenerate electronic states at the Fermi level lead to electronic structures in which correlation and spin play a central role. As a result, charge transport is no longer governed solely by single-particle level alignment but instead reflects the underlying many-body character of the electronic states.

To understand the physics governing charge transport in these emerging molecular platforms, one needs a framework that treats the molecule as a genuinely many-body system and embeds it consistently into external electrodes. In this setting, the transport-relevant charge excitations are no longer appropriately described in terms of canonical molecular orbitals but instead correspond to electron-addition and electron-removal states of the interacting many-body system. Consequently, quantities such as transmission and current must be formulated in terms of many-body Green’s functions and spectral functions, rather than single-particle energy levels and orbital alignments.

To make this many-body perspective concrete while retaining a connection to chemically tunable structure, we consider an interacting one-dimensional model backbone that captures the essential interplay between bond-length alternation (BLA) and electron–electron interactions in open-shell molecular systems. Correlated ground states are obtained using the density matrix renormalization group (DMRG) implemented in the Block2 library,^17^ which provides a nonperturbative and systematically improvable description of quasi-one-dimensional electronic structure.^18–21^ Within this framework, we analyze how dimerization and on-site repulsion control key physical properties, including the spatial distribution of spin density, the range of end-to-end spin correlations, and the singlet–triplet energy splitting. We then compute the interacting Green’s functions of the isolated device using dynamical DMRG (DDMRG), which directly resolves the electron-addition and electron-removal excitations of the correlated system. These are embedded into a nonequilibrium Green’s function (NEGF) framework to describe transport through coupling to external electrodes via lead self-energies, enabling a consistent treatment of the device as a genuinely many-body system under bias. Within this formulation, current is evaluated using the Meir–Wingreen (MW) expression and benchmarked against time-dependent DMRG simulations that directly capture transient dynamics and steady-state transport under finite bias.

Here, we use this framework to examine how BLA and on-site interaction shape the correlated electronic structure and charge transport of open-shell molecular wires. In particular, we focus on how these effects influence the spatial character of low-energy excitations and their contribution to transmission and how this, in turn, controls the dependence of conductance on molecular length. By combining ground-state, spectral, and transport analyses within a unified many-body description, we identify regimes in which transport remains robust over extended distances and relate these behaviors to underlying electronic structure features. This provides a basis for a more systematic physical picture of transport in open-shell molecular backbones and for the development of chemically informed design rules.

To represent a generic open-shell π-conjugated molecular junction, we consider a spinful interacting Su–Schrieffer–Heeger (SSH) backbone embedded within electrodes, as sketched in Figure 1a. The molecular region is described by the device Hamiltonian *H*_dev_:

1 $$H_{\mathrm{dev}}=-\overset{N-1}{\underset{i=1}{\sum }}\underset{\sigma =↑,↓}{\sum }t_{i}\left(c_{i\sigma }^{†}c_{i+1\sigma }+\mathrm{h.c.}\right)+U\overset{N}{\underset{i=1}{\sum }}\left(n_{i↑}-\frac{1}{2}\right)\left(n_{i↓}-\frac{1}{2}\right)$$

where *N* is the number of sites, *c*_*i*_*σ*__^†^ (*c*_*iσ*_) creates (annihilates) an electron with spin σ on site *i*, *n*_*iσ*_ = *c*_*i*_*σ*__^†^ *c*_*iσ*_, and *n*_*i*_ = *n*_*i*__↑_ + *n*_*i*__↓_. The hopping amplitudes *t*_*i*_, which represent the coupling between sites, alternate along the chain (*t*_*i*_ = *t*_1_ for odd *i* and *t*_*i*_ = *t*_2_ for even *i*), and *U* corresponds to the on-site Coulomb repulsion (also known as the Hubbard term), which penalizes double occupancy. The interaction term is written in particle–hole-symmetric form, so that at half-filling and in the absence of site-energy offsets, the model is explicitly particle–hole symmetric. This limit should be viewed as an ideal many-body level alignment reference. In this limit, the electrode Fermi level lies at the symmetry point between the dominant removal and addition resonances. This choice is useful for isolating the roles of bond alternation and electron–electron interaction, but it is not a generic condition for real molecular junctions, where the relative position of resonant transport channels and their broadening depend on anchoring chemistry, contact geometry, molecule–metal charge transfer, interface dipoles, electrostatic gating, and image-charge screening.

**Figure 1:**
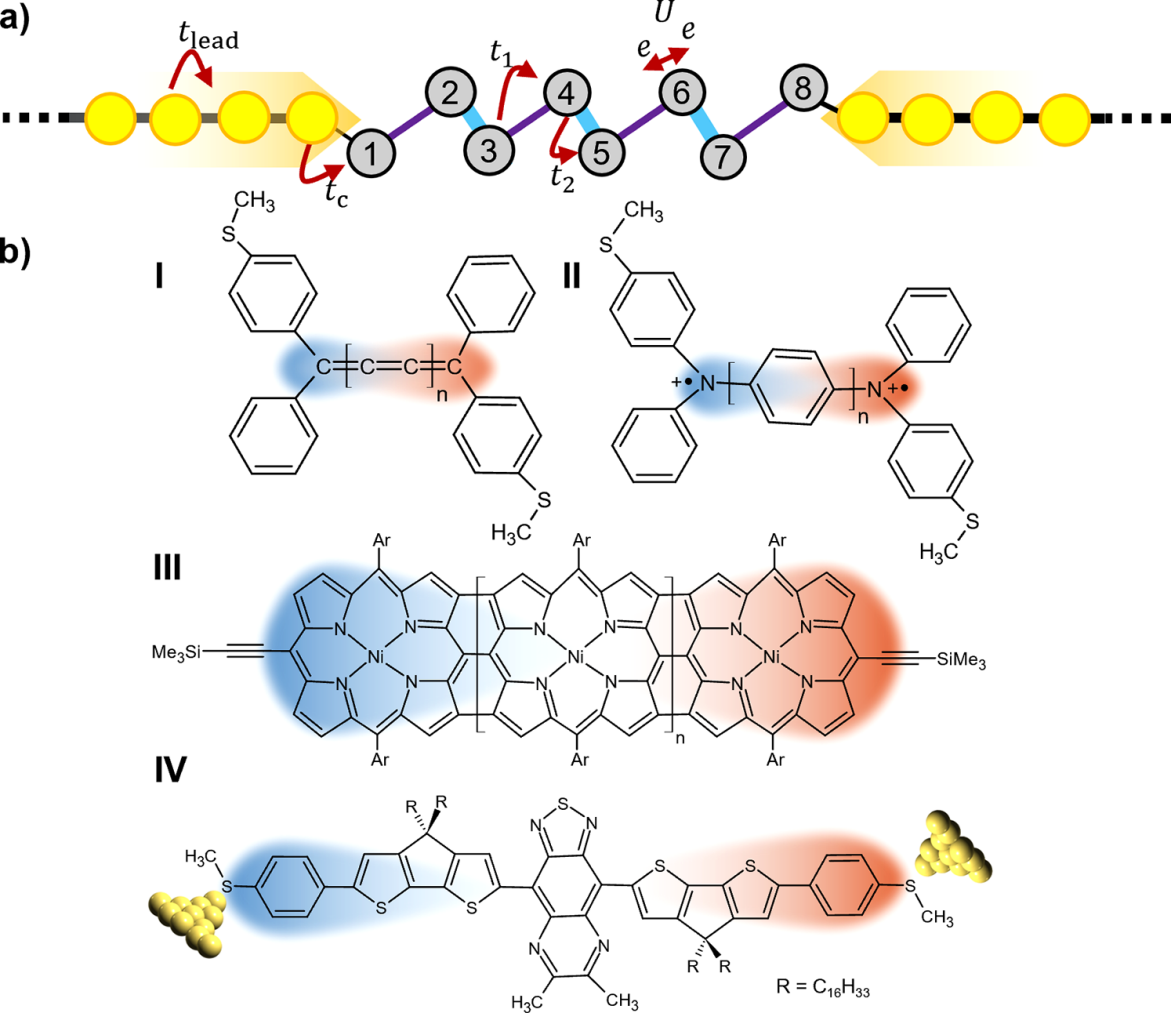
(a) Lead–molecule–lead SSH-Hubbard junction used in this work. A finite dimerized interacting chain with alternating hoppings *t*_1_, *t*_2_, and on-site interaction *U* is contacted by semi-infinite tight-binding leads (hopping *t*_lead_) through a coupling *t*_c_. Site indices label the correlated region. (b) Representative molecular realizations of dimerized backbones: (I) cumulene,^22^ (II) oligophenylene-bridged bis­(triarylamines),^14^ (III) fused porphyrin oligomer,^23^ and (IV) our previously studied DA system.^13^ Blue and red shading indicate opposite signs of the local spin.

Throughout this work, we set ℏ = *e* = 1 unless otherwise stated. All energies are reported in units of *t*_1_. Thus, *U*/*t*_1_, *t*_2_/*t*_1_, *t*_lead_/*t*_1_, *t*_c_/*t*_1_, *E*/*t*_1_, and *V*_bias_/*t*_1_ are dimensionless.

Chemically, *t*_2_/*t*_1_ can be tuned by structural changes that modify π overlap and BLA, including backbone planarity, torsion, ring fusion, and the balance between quinoidal and aromatic resonance structures. Single-molecule conductance studies have shown that torsion suppresses π-conjugation and conductance, while fused or more quinoidal structures can enhance transport by increasing π-delocalization and improving electronic coupling along the backbone.^24,25^ The same parameter is also sensitive to whether the backbone is cumulene-like or polyyne-like, to donor–acceptor patterning, and to cross-conjugation, all of which modify the effective π-coupling pattern and the resulting conductance pathways.^22,26–29^

The interaction parameter *U*/*t*_1_ measures the effective local charging energy relative to that of intramolecular hopping. The ratio increases when the charge and spin densities associated with near-Fermi electronic states are spatially localized on chemically rigid and weakly polarizable units and decreases when these densities are more delocalized and efficiently screened by heteroatoms, polarizable substituents, the surrounding dielectric environment, or nearby electrodes.^1,30^ First-principles Hubbard-parameter studies show that effective *U* values depend sensitively on the chosen localized manifold, local coordination environment, bond geometry, and electronic screening, rather than being universal constants.^31^ In molecular junctions, nearby metallic electrodes can also renormalize molecular addition and removal energies through polarization and image-charge screening.^32,33^

Finally, *t*_c_/*t*_1_ describes the effective molecule–electrode coupling relative to intramolecular hopping. In molecular junctions, this coupling is controlled by the anchoring group, metal identity, local contact geometry, linker conjugation, and molecule–electrode hybridization. Experiments and calculations have shown that anchoring groups can strongly change single-molecule conductance and that different molecule–electrode binding geometries can produce distinct conductance states or stretching-dependent conductance changes.^34–36^

For a chemically specific molecular junction, these effective parameters can in principle be obtained by downfolding a first-principles calculation of the relaxed junction geometry into a localized active π-electron basis, for example using maximally localized Wannier functions.^37,38^ The alternating nearest-neighbor matrix elements of the projected one-electron Hamiltonian then define *t*_2_/*t*_1_. The effective on-site interaction *U* must be evaluated on that same localized basis and represents the on-site Coulomb interaction. In constrained or linear-response DFT, *U*/*t*_1_ is extracted from the difference between the bare and self-consistently screened occupation responses to local potential shifts in the active subspace, *U*_*i*_ = [χ_0_^–1^ – χ^–1^]_*ii*_.^30^ Density-functional perturbation theory provides an equivalent route, while constrained random-phase approximation computes a partially screened interaction after excluding polarization processes internal to the active π-electron space.^31,39^ The contact parameter *t*_c_/*t*_1_ can be obtained by projecting the molecule–electrode coupling onto the terminal active orbitals or, within the present effective lead representation, by matching the projected lead self-energy and associated broadening Γ_L,R_(*E*) near the transport-relevant resonances. The resulting parameters are therefore specific to the selected active basis and relaxed junction geometry.

Figure 1b exemplifies how some representative junctions, such as cumulenes (*t*_2_ ≈ *t*_1_), fused porphyrins (*t*_2_ ≫ *t*_1_), and diradical donor–acceptor wires, are mapped into this interacting model, which we refer to as the spinful SSH-Hubbard model.^13,22,23,26^

We begin by analyzing the many-body ground-state properties of the isolated molecule, as they establish the correlated spin and charge distributions that set the energy scales and spatial structure of the excitations governing transport. To do so, we performed DMRG calculations (Figures S1 and S2) on the spinful SSH-Hubbard backbone with open ends, as described by eq 1.

Figure 2 shows the spin *S*_*z*_ projection of the isolated backbone for two chain lengths, *N* = 20 and 8, as a function of BLA and on-site interaction. For fixed dimerization (Figure 2a,b), increasing *U*/*t*_1_ primarily amplifies the staggered component of the spin texture rather than changing its overall decay length. The termini retain the same dominant sign of *S*_*z*_, but small opposite-sign lobes appear and grow in amplitude in an alternating fashion around the ends. In other words, the edge polarization becomes more staggered with *U*/*t*_1_, while the envelope that sets how fast the signal decays into the interior remains nearly unchanged over the *U*/*t*_1_ range examined. This pattern is consistent with stronger short-range antiferromagnetic correlations superimposed on a similar spatial envelope (Figure S3).^40^

**Figure 2:**
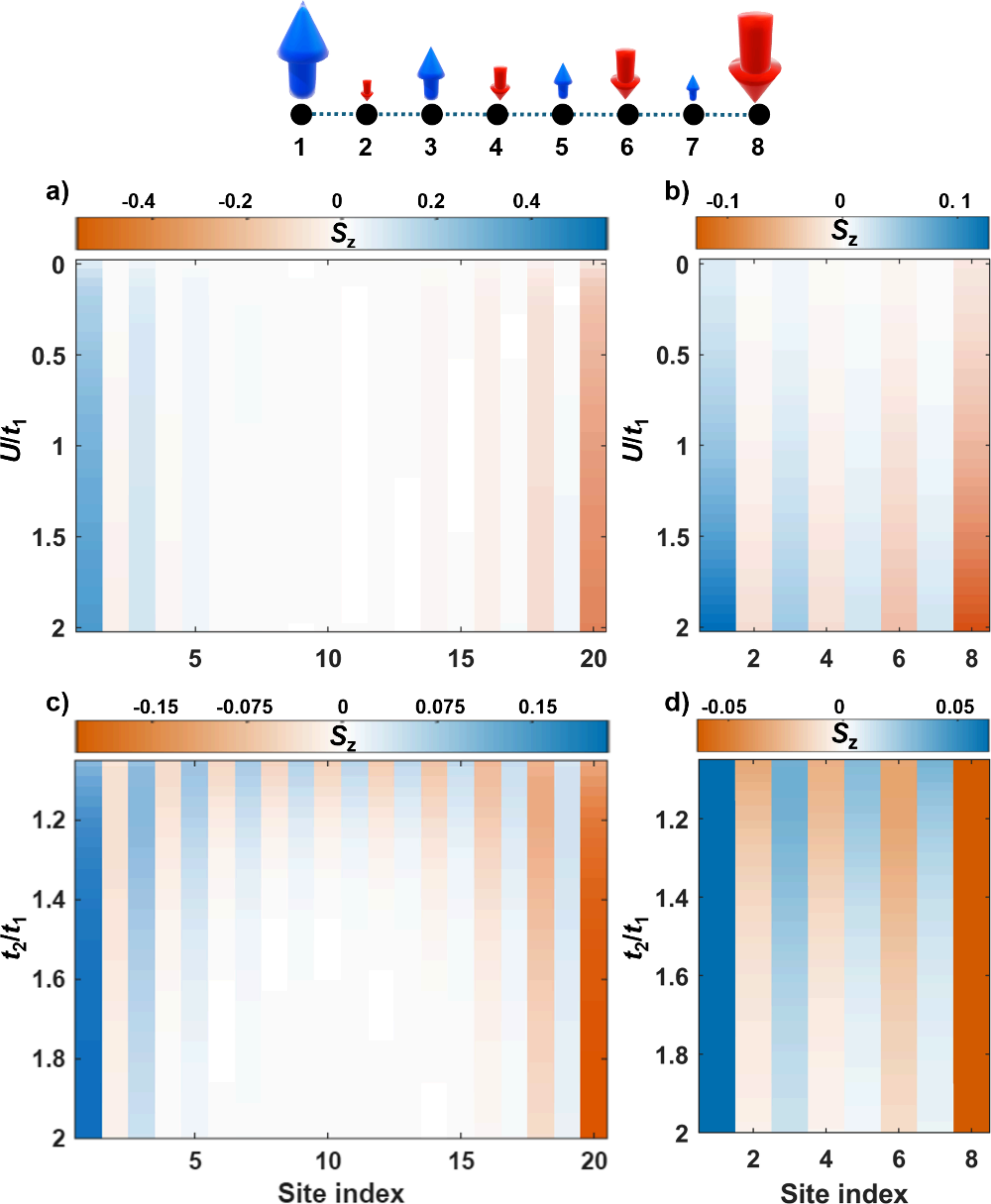
Spin density *S*_*z*_ evolution map as *U*/*t*_1_ increases and *t*_2_/*t*_1_ = 1.5 is fixed for (a) *N* = 20 and (b) *N* = 8. The *S*_*z*_ evolution map as *t*_2_/*t*_1_ increases and the *U*/*t*_1_ = 2 is fixed for (c) *N* = 20 and (d) *N* = 8.

Dimerization (*t*_2_/*t*_1_) primarily controls the spatial extent of the edge states (Figure 2c,d) and therefore the range over which spin and charge correlations propagate. In the bulk SSH limit, tuning *t*_2_/*t*_1_ crosses between topological (*t*_2_ > *t*_1_) and trivial (*t*_2_ < *t*_1_) gapped phases, with a gap closing critical point at *t*_2_ = *t*_1_.^41,42^ For an open chain this distinction is reflected in the presence or absence of exponentially localized edge states. For the noninteracting SSH model in the topological regime *t*_2_ > *t*_1_, the edge-state localization length scales approximately as ξ ∼ [ln­(*t*_2_/*t*_1_)]^−1^.^16^ Consistent with this, in the interacting SSH-Hubbard, the DMRG results show that increasing *t*_2_/*t*_1_ also compresses the envelope of *S*_*z*_ toward the termini (stronger edge localization), while decreasing *t*_2_/*t*_1_ extends it. Superimposed on this envelope, the *U*-induced staggering discussed above persists, but the range over which staggered oscillations are visible is set primarily by *t*_2_/*t*_1_.

These trends already reveal how interaction and dimerization play distinct roles: while *U*/*t*_1_ primarily controls the local spin correlations, BLA determines the spatial extent over which these correlations propagate.

The changes in the edge-state localization are also reflected in the singlet–triplet splitting Δ_ST_ (Figure 3), which provides a direct measure of the effective exchange *J*_eff_ between the two terminal spins and, therefore, of the degree of hybridization between the edge-localized states. Increasing *t*_2_/*t*_1_ localizes the edge modes and exponentially reduces their overlap across the backbone, strongly suppressing *J*_eff_ and hence |Δ_ST_| (Figure S4). Increasing *U*/*t*_1_ suppresses |Δ_ST_|/*t*_1_ for a different reason: it penalizes the virtual charge fluctuations (double occupancy) that mediate the edge–edge coupling, thereby reducing the superexchange scale. Specifically, *J*_eff_ ∝ *t*_eff_^2^/*U* where *t*_eff_ denotes the effective hybridization between the two edge-localized states set by their finite overlap across the backbone.^43–45^

**Figure 3:**
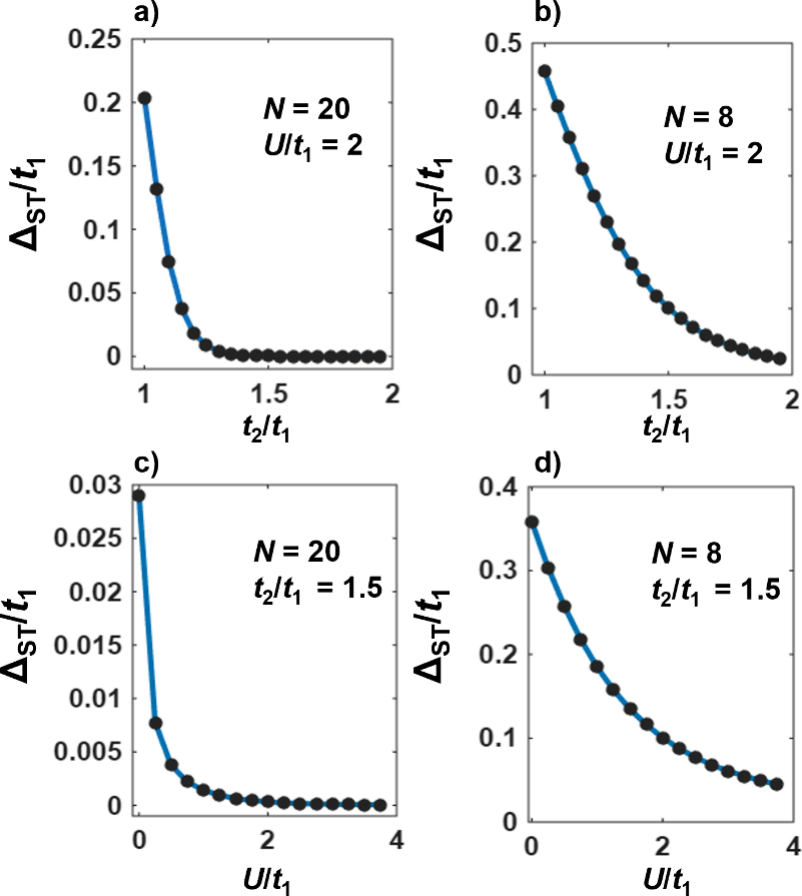
Singlet–triplet energy gap Δ_ST_/*t*_1_ vs *t*_2_/*t*_1_ obtained from DMRG for (a) *N* = 20 and (b) *N* = 8 where *U*/*t*_1_ = 2 is fixed. Δ_ST_/*t*_1_ vs *U*/*t*_1_ for (c) *N* = 20 and (d) *N* = 8; *t*_2_/*t*_1_ = 1.5 is fixed here.

The ground-state results already suggest clear design principles for open-shell molecular wires. First, reducing BLA (driving *t*_2_/*t*_1_ → 1) maintains spatially extended edge states that remain hybridized across long backbones, even for very long molecules. Second, a finite on-site interaction (0.5 < *U*/*t*_1_ < 1.5 in our model) stabilizes local moments and enhances edge polarization. However, excessively large *U*/*t*_1_ suppresses the charge fluctuations required for transport. As we show below, optimal transport arises from a balance between these effects.

Recent reports of unusually high conductance in open-shell π-conjugated junctions, including backbones with substantial diradical character, highlight that electron–electron interactions can be a central part of the transport physics rather than a minor correction to a one-electron picture.^13,14,23^ In these systems, reduced screening increases the energy cost of adding or removing charge, and electronic states near the Fermi level can lie close in energy and support different spin configurations. As a result, low-bias transport is governed by which correlated electron-addition and electron-removal excitations are energetically accessible. Single-determinant and mean-field treatments can therefore become unreliable because they do not represent these correlated excitations correctly, leading to incorrect energies and intensities of the transport-relevant features that couple to the electrodes. Capturing these effects is computationally demanding, but it is precisely what is required to obtain predictive, chemically actionable design rules for correlated molecular quantum devices.^46,47^

To understand how the correlated electronic structure identified above translates into transport, we adopt a hybrid approach in which the many-body response of the molecule is obtained from DDMRG, while its coupling to external electrodes is treated within the NEGF formalism. In a correlated system, transport is governed not by single-particle orbitals but by electron-addition and electron-removal excitations. These excitations are encoded in the retarded Green’s tensor of the isolated backbone, *G*_0,*ij*,σ_^*r*^(ω), which resolves the frequency-dependent response between sites *i* and *j*. The isolated many-body Green’s tensor is then embedded into a lead–molecule–lead junction through electrode self-energies. In this construction, the molecular backbone remains correlated through the DDMRG Green’s function, whereas the electrodes are treated as noninteracting reservoirs that impose broadening and nonequilibrium occupations. The contacted retarded and advanced Green’s functions are obtained by Dyson embedding, and the lesser component is constructed using the Kadanoff–Baym relation so that both the correlated molecular occupation and the bias-driven lead populations are retained. The steady-state current is then evaluated using the MW expression, with the full formulation given in the Supporting Information (see detailed discussion in Section S2 and Figures S5 and S6).

Figure 4 shows the evolution of correlated transport as a function of interaction strength, dimerization, and system size. For the dimerized chain with *N* = 8 and *t*_2_/*t*_1_ = 1.5, the two near-zero-energy features at *U*/*t*_1_ = 0 originate from the finite overlap between the edge-localized states, which lifts their degeneracy and produces a small midgap splitting. In the many-body spectrum, this splitting appears as a pair of closely spaced electron-addition and electron-removal excitations, giving rise to the corresponding pair of transmission resonances near the Fermi level (Figure 4a).

**Figure 4:**
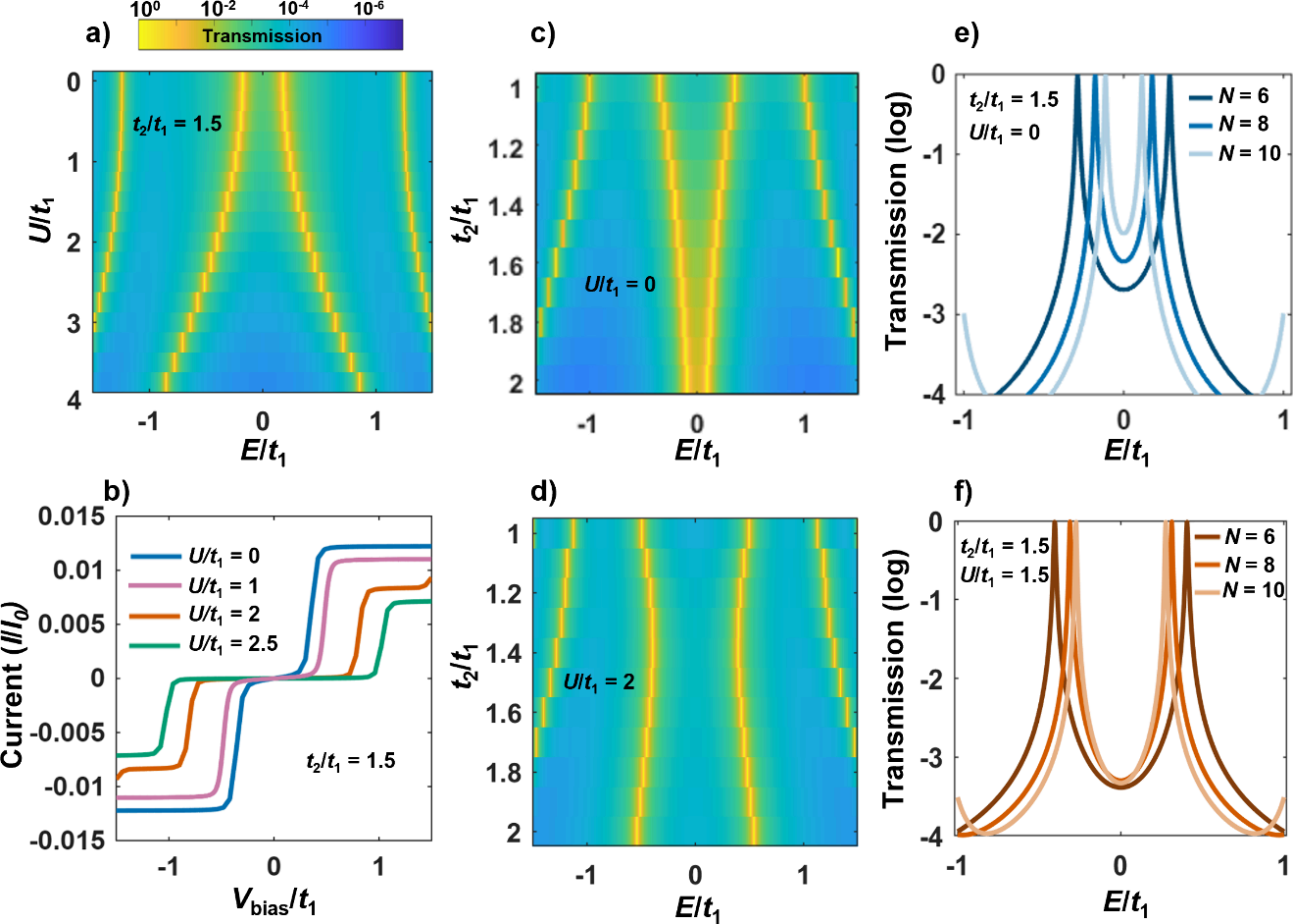
Correlated transport as a function of interaction strength, dimerization, and system size. (a) Transmission (MW integrand) as a function of energy for increasing *U*/*t*_1_ at fixed *N* = 8 and *t*_2_/*t*_1_ = 1.5. (b) Corresponding current–voltage (*I*–*V*) characteristics for the system in (a) at different values of *U*/*t*_1_. (c) Transmission as a function of energy for increasing *t*_2_/*t*_1_ at fixed *N* = 8 and *U*/*t*_1_ = 0. (d) Transmission as a function of energy for increasing *t*_2_/*t*_1_ at fixed *N* = 8 and *U*/*t*_1_ = 2. (e) Transmission for different chain lengths *N* = 6, 8, and 10 at fixed *U*/*t*_1_ = 0 and *t*_2_/*t*_1_ = 1.5. (f) Transmission for *N* = 6, 8, and 10 at fixed *U*/*t*_1_ = 1.5 and *t*_2_/*t*_1_ = 1.5. For all panels, the leads are semi-infinite tight-binding chains with nearest-neighbor hopping *t*_lead_/*t*_1_ = 1 and lead–device coupling *t*_c_/*t*_1_ = 0.1. Current is reported in units of *I*_0_ = *et*_1_/ℏ. In our numerical units, *e* = ℏ = 1.

When an on-site interaction is introduced, the transport features reorganize because the relevant low-energy addition and removal excitations are predominantly localized at the chain ends. Although the Hubbard interaction acts on every site, these midgap excitations have their largest weight on the terminal sites, making them particularly sensitive to *U*. At half-filling and in the presence of particle–hole symmetry, the correlated ground state develops one singly occupied edge-localized state at each end, corresponding to an effective spin-1/2 moment per terminus. As *U*/*t*_1_ increases, the dominant removal excitation corresponds to emptying a singly occupied edge state, while the dominant addition excitation creates a doubly occupied edge state and, therefore, incurs an interaction energy cost. As a result, the corresponding resonances move symmetrically away from the Fermi level, and the MW integrand evolves into a split pair of peaks centered at zero energy.

At fixed interaction *U*/*t*_1_ = 0, increasing the dimerization drives the two midgap resonances toward each other until they merge (Figure 4c). In the SSH backbone, the splitting between the edge states is controlled by their hybridization across the chain. As *t*_2_/*t*_1_ increases, the bulk gap widens and the edge-state localization length ξ decreases, leading to an exponential suppression of their overlap and, consequently, of the midgap splitting. At finite interaction (*U*/*t*_1_ = 2), the evolution with dimerization becomes nonmonotonic due to the competition between hybridization and interaction effects (Figure 4d, Figure S7). For moderate *t*_2_/*t*_1_, the transmission peaks near zero energy are still governed primarily by edge-state hybridization and move closer together as this overlap is reduced. However, as the edge states become more strongly localized, the corresponding charge excitations become increasingly confined to the terminal sites, enhancing the effective interaction they experience. This leads to an increased separation between the addition and removal resonances. Consequently, beyond a certain dimerization strength, the interaction-driven splitting dominates, and the two peaks move apart.

Figure 4b shows the corresponding current–voltage characteristics for *t*_2_/*t*_1_ = 1.5 at several interaction strengths *U*, obtained from the MW current expression (eq S15). The evolution of the low-energy spectral features directly controls the onset and shape of the current. Figure 4e and 4f examine dependence on system size. At *U*/*t*_1_ = 0 (Figure 4e), increasing *N* reduces the overlap between the edge states, causing the two near-zero-energy resonances to approach each other and eventually merge, leading to an enhanced transmission at the Fermi level. In contrast, at finite moderate interaction (*U*/*t*_1_ = 1.5, Figure 4f), the separation between addition and removal excitations is partially maintained even as the edge-state overlap decreases. As a result, the transmission profile near zero energy evolves much more weakly with system size. This slower collapse of the low-energy spectral features suggests that intermediate interaction strengths (0.5 < *U*/*t*_1_ < 1.5) can stabilize transport channels over longer distances, providing a favorable regime for long-range charge transport.

While the DDMRG+MW framework provides steady-state currents, it is important to validate these results using a fully time-dependent approach that does not rely on equilibrium assumptions. In the MW-NEGF framework, the current depends explicitly on the nonequilibrium lesser Green’s function *G* ^<^(ω) of the contacted device. Once a bias is applied, the equilibrium fluctuation–dissipation relation no longer determines the occupations of the contacted system. Instead *G* ^<^(ω) must be obtained from the Kadanoff–Baym equations using the lead self-energies and the nonequilibrium lead distributions. For moderate (0.5 < *U*/*t*_1_ < 1.5) interactions, the approach to steady state can additionally involve bias-driven charge redistribution and correlation buildup near the contacts, which motivates an independent check of the steady current.

We therefore complement the frequency-domain MW currents with real-time tDMRG, which evolves the full contacted Hamiltonian under a smooth bias ramp and evaluates the current operator directly without invoking equilibrium closure relations for nonequilibrium occupations. This provides a stringent benchmark for the MW embedding in the parameter regime studied and additionally gives access to dynamical information not available in the frequency domain, including the charging transient, the relaxation into a quasi-steady current plateau, and the accompanying time-dependent redistribution of spin density.^48–50^

We adopt a partition-free protocol in which the full finite junction is first prepared in its interacting ground state at half-filling, and the bias is then switched on smoothly using a *tanh* ramp to minimize quench artifacts. Time evolution is carried out with a matrix product state implementation of the two-site time-dependent variational principle (TDVP) as implemented in the Block2 package, using a time step Δ*t* = 0.05, truncation tolerance ϵ = 10^–8^, and a capped evolution bond dimension χ_evo_ = 1024, with convergence verified against these parameters (Figure S10).^51,52^

During the evolution, we monitor bond currents at the two contacts, *I*_L_(*t*) and *I*_R_(*t*), computed from the standard lattice current operator on the lead–device bond *Ĵ*_*i*__,*i*+1_ = *it*_*i*__,*i*+1_∑_σ_(*c*_*i*_σ__^†^ *c*_*i*__+1,σ_ – h.c.) together with spin-resolved site occupations *n*_↑,↓_(*i*,*t*) (Figure S11). A quasi-steady current is extracted by time-averaging over the plateau window that forms after the initial charging transient and before boundary reflections from the finite leads’ return to the device.

We consider a central region with *N* = 8 sites coupled to left and right leads of *N*_lead_ = 80 sites each, with *t*_lead_/*t*_1_ = 1 and device–lead coupling *t*_c_/*t*_1_ = 0.1. Representative current traces (Figure 5a,c,e) show a short charging transient following the bias ramp, followed by damped oscillations that decay into a quasi-steady plateau before finite-size reflections occur.

**Figure 5:**
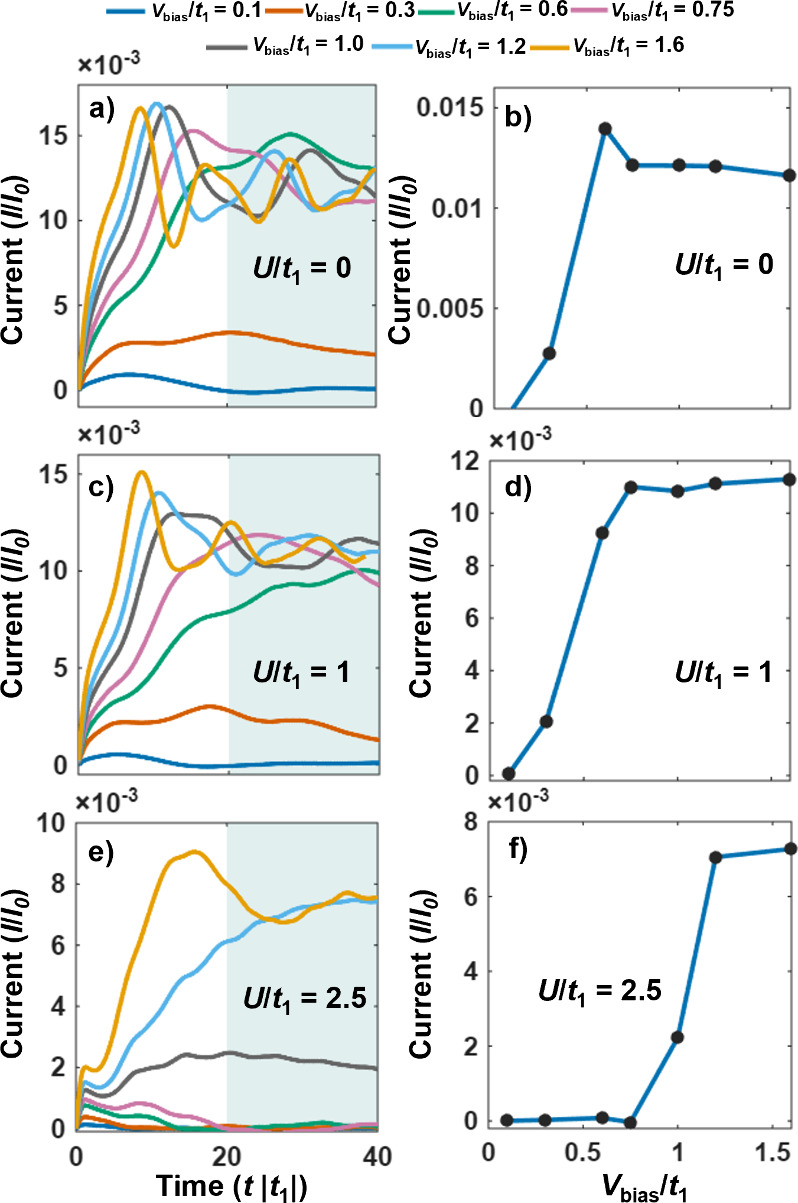
Time-dependent DMRG current vs time results and the corresponding *I*–*V* curve from a steady state current plateau for (a, b) *U*/*t*_1_ = 0, (c, d) *U*/*t*_1_ = 1, and (e, f) *U*/*t*_1_ = 2.5. The shaded area corresponds to the time window that the currents are averaged and later indicated as a point in the *I*–*V* plots. In these calculations *N* = 8, *N*_lead_ = 80, *t*_lead_/*t*_1_ = 1, *t*_c_/*t*_1_ = 0.1, and *t*_2_/*t*_1_ = 1.5. Current is reported in units of *I*_0_ = *et*_1_/ℏ. In our numerical units, *e* = ℏ = 1. We monitor bond currents at the two contacts, *I*_L_ and *I*_R_, so that charge conservation in the quasi-steady plateau gives *I*_L_ ≈ −*I*_R_. The plotted current is the symmetrized junction current, *I* = (*I*_L_ – *I*_R_)/2.

The resulting *I*–*V* curves (Figure 5b,d,f) are in good agreement with the frequency-domain currents shown in Figure 4b, obtained using the same Hamiltonian and lead parameters. This consistency confirms that the MW embedding captures the essential steady-state transport physics in the parameter regimes studied.

As *U*/*t*_1_ increases, the transient current rises more gradually and exhibits a reduced overshoot before reaching a quasi-steady plateau. This behavior can be directly traced to the correlated spectral features identified in Figure 4. Increasing *U*/*t*_1_ separates the near-zero-energy electron addition and electron removal resonances, thereby reducing the spectral weight within the bias window set by the leads. As a result, fewer transport channels are energetically accessible immediately after the bias ramp, leading to less abrupt carrier injection and a slower buildup of current.

In this work, we established a physically transparent framework for understanding charge transport in chemically motivated models of open-shell π-conjugated molecular systems by combining many-body electronic structure with nonequilibrium transport theory. Our results show that transport in these systems is governed by edge-localized many-body excitations, rather than by single-particle molecular orbitals, and therefore requires an explicitly correlated description.

The analysis reveals two key microscopic parameters that control the transport. BLA determines the spatial extent and hybridization of edge states, while on-site interactions set the energetic splitting between addition and removal excitations. Together, these parameters define a regime in which edge states remain both spatially extended and energetically accessible within the bias window, enabling efficient charge transport over ultralong molecular backbones.

These findings lead to clear design principles for highly transmissive molecular conductors: minimizing BLA promotes long-range delocalization, while maintaining a finite but moderate interaction strength (0.5 < *U*/*t*_1_ < 1.5 in our model) stabilizes open-shell character without suppressing transport. Optimal conductance emerges from a balance between these effects, where neither strong localization nor excessive correlation dominates. Donor–acceptor molecular architectures provide especially attractive platforms to realize this regime, as both dimerization and effective interaction can be tuned chemically through backbone patterning, conjugation, spin-density localization, and dielectric screening.

More broadly, this work highlights the limitations of single-particle transport approaches in systems with near degeneracy and spin polarization and demonstrates that many-body effects can be harnessed to design molecular-scale conductors with unconventional transport properties. By establishing a direct connection between chemically controlled molecular structure and correlated transport behavior, the framework developed here provides a practical route toward the predictive design of open-shell molecular electronic devices beyond mean-field descriptions.

## Supplementary Material



## Notes

Details of the calculation and convergence tests (PDF)

## References

[1] Evers F., Korytár R., Tewari S., van Ruitenbeek J. M. (2020). Advances
and challenges in single-molecule electron transport. Rev. Mod. Phys..

[2] Jia C., Migliore A., Xin N., Huang S., Wang J., Yang Q., Wang S., Chen H., Wang D., Feng B. (2016). Covalently
bonded single-molecule junctions with stable
and reversible photoswitched conductivity. Science.

[3] Guo C., Wang K., Zerah-Harush E., Hamill J., Wang B., Dubi Y., Xu B. (2016). Molecular
rectifier composed of DNA
with high rectification ratio enabled by intercalation. Nat. Chem..

[4] Meng L., Xin N., Hu C., Sabea H. A., Zhang M., Jiang H., Ji Y., Jia C., Yan Z., Zhang Q. (2022). Dual-gated
single-molecule field-effect transistors beyond Moore’s law. Nat. Commun..

[5] Vincent R., Klyatskaya S., Ruben M., Wernsdorfer W., Balestro F. (2012). Electronic read-out
of a single nuclear spin using
a molecular spin transistor. Nature.

[6] Wagner S., Kisslinger F., Ballmann S., Schramm F., Chandrasekar R., Bodenstein T., Fuhr O., Secker D., Fink K., Ruben M. (2013). Switching of a coupled spin pair in a single-molecule
junction. Nat. Nanotechnol..

[7] Moreno-Pineda E., Godfrin C., Balestro F., Wernsdorfer W., Ruben M. (2018). Molecular spin qudits for quantum
algorithms. Chem. Soc. Rev..

[8] Nitzan A., Ratner M. A. (2003). Electron transport
in molecular wire junctions. Science.

[9] Brandbyge M., Mozos J.-L., Ordejón P., Taylor J., Stokbro K. (2002). Density-functional
method for nonequilibrium electron transport. Phys. Rev. B.

[10] Taylor J., Guo H., Wang J. (2001). Ab initio
modeling of quantum transport properties
of molecular electronic devices. Phys. Rev.
B.

[11] Datta, S. Quantum Transport: Atom to Transistor; Cambridge University Press, 2005, 10.1017/CBO9781139164313.

[12] Mejía L., Kleinekathöfer U., Franco I. (2022). Coherent and
incoherent
contributions to molecular electron transport. J. Chem. Phys..

[13] Shen S., Shiri M., Mahalingam P., Tang C., Bills T., Bushnell A. J., Balandin T. A., Mejía L., Zhang H., Xu B. (2025). Long-range
resonant
charge transport through open-shell donor-acceptor macromolecules. J. Am. Chem. Soc..

[14] Li L., Low J. Z., Wilhelm J., Liao G., Gunasekaran S., Prindle C. R., Starr R. L., Golze D., Nuckolls C., Steigerwald M. L. (2022). Highly conducting single-molecule topological
insulators based on mono- and di-radical cations. Nat. Chem..

[15] Li L., Prindle C. R., Shi W., Nuckolls C., Venkataraman L. (2023). Radical single-molecule
junctions. J. Am. Chem. Soc..

[16] Li L., Gunasekaran S., Wei Y., Nuckolls C., Venkataraman L. (2022). Reversed conductance
decay of 1D topological insulators by tight-binding analysis. J. Phys. Chem. Lett..

[17] Zhai H., Larsson H. R., Lee S., Cui Z.-H., Zhu T., Sun C., Peng L., Peng R., Liao K., Tölle J. (2023). Block2: a comprehensive open source framework
to develop and apply
state-of-the-art DMRG algorithms in electronic structure and beyond. J. Chem. Phys..

[18] Chan G. K.-L., Head-Gordon M. (2002). Highly correlated
calculations with a polynomial cost
algorithm: a study of the density matrix renormalization group. J. Chem. Phys..

[19] Östlund S., Rommer S. (1995). Thermodynamic limit of density matrix renormalization. Phys. Rev. Lett..

[20] White S. R. (1992). Density
matrix formulation for quantum renormalization groups. Phys. Rev. Lett..

[21] White S. R. (1993). Density-matrix
algorithms for quantum renormalization groups. Phys. Rev. B.

[22] Zang Y., Fu T., Zou Q., Ng F., Li H., Steigerwald M. L., Nuckolls C., Venkataraman L. (2020). Cumulene wires display increasing
conductance with increasing length. Nano Lett..

[23] Deng J.-R., González M. T., Zhu H., Anderson H. L., Leary E. (2024). Ballistic
conductance through porphyrin nanoribbons. J.
Am. Chem. Soc..

[24] Venkataraman L., Klare J. E., Nuckolls C., Hybertsen M. S., Steigerwald M. L. (2006). Dependence of single-molecule junction conductance
on molecular conformation. Nature.

[25] Chen W., Li H., Widawsky J. R., Appayee C., Venkataraman L., Breslow R. (2014). Aromaticity decreases
single-molecule junction conductance. J. Am.
Chem. Soc..

[26] Xu W., Leary E., Hou S., Sangtarash S., González M. T., Rubio-Bollinger G., Wu Q., Sadeghi H., Tejerina L., Christensen K. E. (2019). Unusual length dependence
of the conductance in cumulene molecular wires. Angew. Chem., Int. Ed..

[27] Nacci C., Ample F., Bleger D., Hecht S., Joachim C., Grill L. (2015). Conductance of a single flexible molecular wire composed of alternating
donor and acceptor units. Nat. Commun..

[28] Valkenier H., Guédon C. M., Markussen T., Thygesen K. S., van der
Molen S. J., Hummelen J. C. (2014). Cross-conjugation and quantum interference:
a general correlation?. Phys. Chem. Chem. Phys..

[29] Solomon G. C., Andrews D. Q., Goldsmith R. H., Hansen T., Wasielewski M. R., Van Duyne R. P., Ratner M. A. (2008). Quantum interference in acyclic systems:
conductance of cross-conjugated molecules. J.
Am. Chem. Soc..

[30] Cococcioni M., de Gironcoli S. (2005). Linear response approach to the calculation of the
effective interaction parameters in the LDA+U method. Phys. Rev. B.

[31] Bastonero L., Malica C., Macke E., Bercx M., Huber S., Timrov I., Marzari N. (2025). First-principles Hubbard parameters
with automated and reproducible workflows. npj
Comput. Mater..

[32] Sakuma R., Aryasetiawan F. (2013). First-principles
calculations of dynamical screened
interactions for the transition metal oxides MO (M = Mn, Fe, Co, Ni). Phys. Rev. B.

[33] Neaton J. B., Hybertsen M. S., Louie S. G. (2006). Renormalization of molecular electronic
levels at metal-molecule interfaces. Phys. Rev.
Lett..

[34] Chen F., Li X., Hihath J., Huang Z., Tao N. (2006). Effect of anchoring
groups on single-molecule conductance: comparative study of thiol-,
amine-, and carboxylic-acid-terminated molecules. J. Am. Chem. Soc..

[35] Kamenetska M., Quek S. Y., Whalley A. C., Steigerwald M. L., Choi H. J., Louie S. G., Nuckolls C., Hybertsen M. S., Neaton J. B., Venkataraman L. (2010). Conductance and geometry of pyridine-linked
single-molecule junctions. J. Am. Chem. Soc..

[36] Kim Y.-H., Kim H. S., Lee J., Tsutsui M., Kawai T. (2017). Stretching-induced
conductance variations as fingerprints of contact configurations in
single-molecule junctions. J. Am. Chem. Soc..

[37] Chang Y., van Loon E. G. C. P., Eskridge B., Busemeyer B., Morales M. A., Dreyer C. E., Millis A. J., Zhang S., Wehling T. O., Wagner L. K. (2024). Downfolding from ab
initio to interacting model Hamiltonians: comprehensive analysis and
benchmarking of the DFT+cRPA approach. npj Comput.
Mater..

[38] Mostofi A. A., Yates J. R., Pizzi G., Lee Y.-S., Souza I., Vanderbilt D., Marzari N. (2014). An updated version of wannier90:
a tool for obtaining maximally-localised Wannier functions. Comput. Phys. Commun..

[39] Timrov I., Marzari N., Cococcioni M. (2018). Hubbard parameters
from density-functional
perturbation theory. Phys. Rev. B.

[40] Essler, F. H. L. ; Frahm, H. ; Göhmann, F. ; Klümper, A. ; Korepin, V. E. The One-Dimensional Hubbard Model; Cambridge University Press, 2005, 10.1017/CBO9780511534843.

[41] Asbóth, J. K. ; Oroszlány, L. ; Pályi, A. A Short Course on Topological Insulators: Band Structure and Edge States in One and Two Dimensions; Lecture Notes in Physics; Springer, 2016, 10.1007/978-3-319-25607-8.

[42] Su W. P., Schrieffer J. R., Heeger A. J. (1980). Soliton excitations in polyacetylene. Phys. Rev. B.

[43] Anderson P. W. (1950). Antiferromagnetism.
Theory of superexchange interaction. Phys. Rev..

[44] MacDonald A. H., Girvin S. M., Yoshioka D. (1988). *t*/*U* expansion for the Hubbard model. Phys. Rev.
B.

[45] Auerbach, A. Interacting Electrons and Quantum Magnetism; Graduate Texts in Contemporary Physics; Springer, 1994, 10.1007/978-1-4612-0869-3.

[46] Koentopp M., Burke K., Evers F. (2006). Zero-bias molecular electronics:
exchange-correlation corrections to Landauer’s formula. Phys. Rev. B.

[47] Liang W., Shores M. P., Bockrath M., Long J. R., Park H. (2002). Kondo resonance
in a single-molecule transistor. Nature.

[48] Heidrich-Meisner F., Feiguin A. E., Dagotto E. (2009). Real-time
simulations of nonequilibrium
transport in the single-impurity Anderson model. Phys. Rev. B.

[49] White S. R., Feiguin A. E. (2004). Real-time evolution
using the density matrix renormalization
group. Phys. Rev. Lett..

[50] Schmitteckert P. (2004). Nonequilibrium
electron transport using the density matrix renormalization group
method. Phys. Rev. B.

[51] Haegeman J., Cirac J. I., Osborne T. J., Pižorn I., Verschelde H., Verstraete F. (2011). Time-dependent variational principle
for quantum lattices. Phys. Rev. Lett..

[52] Haegeman J., Lubich C., Oseledets I., Vandereycken B., Verstraete F. (2016). Unifying time evolution and optimization
with matrix
product states. Phys. Rev. B.

