# Supplementary material for: Design Rules for Open-Shell Molecular Wires: Insights from Correlated Many-Body Transport
Source: Nano Lett. 2026 Jul 27;26(31):10256–63. doi: 10.1021/acs.nanolett.6c02096 (PMC13475042; doi:10.1021/acs.nanolett.6c02096)
Supplement: Supplementary file 1 [file nl6c02096_si_001.pdf]

Supporting Information

Design Rules for Open-Shell Molecular Wires: Insights from Correlated Many-Body Transport

Mehrdad Shiri[1], Leopoldo Mejía[2], Ignacio Franco[3,4,5], Thorsten Hansen[6*], Kun Wang[1,7*]*

[1]Department of Physics, University of Miami, Coral Gables, FL 33146, United States.

[2]Departamento de Física y Astronomía, Facultad de Ciencias Exactas, Universidad Andres Bello, Santiago 837-0136, Chile.

[3]Department of Chemistry, University of Rochester, Rochester, NY 14627, United States.

[4]Department of Physics and Astronomy, University of Rochester, Rochester, NY 14627, United States.

[5]Institute of Optics, University of Rochester, Rochester, NY 14627, United States.

[6]Department of Chemistry, University of Copenhagen, DK 2100 Copenhagen Ø, Denmark.

[7]Department of Chemistry, University of Miami, Coral Gables, FL 33146, United States.

*Correspondence should be addressed to Ignacio Franco (ignacio.franco@rochester.edu), Thorsten Hansen (thorsten@chem.ku.dk), and Kun Wang (kunwang@miami.edu).

Supporting Information - Table of Contents

S1. Static DMRG (Ground-State Calculations)

S1.1 Model Hamiltonian and conventions

S1.2 DMRG setup and convergence tests (bond dimension, sweeps, truncation error, energy convergence)

S1.3 Ground-state observables

S2. Dynamical DMRG (DDMRG) and Meir-Wingreen Transport

S2.1 Retarded Green's tensor in correction-vector DDMRG

S2.2 Hubbard dimer benchmark

S2.3 Broadening dependence of the MW integrand (effective transmission)

S2.4 Trivial *vs* topological regime control calculations for the SSH-Hubbard chain

S2.5 Introducing a staggered donor-acceptor onsite potential

S2.6 Mean-field Landauer reference calculations

S3. Time-Dependent DMRG Under Finite Bias

S3.1 Junction geometry for time evolution (finite leads, couplings)

S3.2 Bias protocol (*tanh* ramp) and quench mitigation

S3.3 Time-evolution parameters (Δt, cutoff, χ_{evo})

S1. Static DMRG (Ground-State Calculations)

S1.1 Model Hamiltonian and Conventions

The ground-state calculations in this work are performed for a finite, open-boundary spinful SSH-Hubbard segment of length L, described by the device Hamiltonian

$$H_{\text{dev}} = -\sum_{i=1}^{N-1}\sum_{\sigma=\uparrow,\downarrow} t_i\left(c_{i\sigma}^{\dagger}c_{i+1\sigma}+\text{h.c.}\right)+U\sum_{i=1}^{N}\left(n_{i\uparrow}-\frac{1}{2}\right)\left(n_{i\downarrow}-\frac{1}{2}\right) \quad \text{(S1)}$$

with alternating nearest neighbor hoppings $t_i \in t_1, t_2$ and on-site repulsion U. Here $c_{i\sigma}^{\dagger}(c_{i\sigma})$ creates (annihilates) an electron of spin σ on site i, $n_{i\sigma} = c_{i\sigma}^{\dagger}c_{i\sigma}$, and $n_i = n_{i\uparrow} + n_{i\downarrow}$. We use open boundary conditions throughout. The interaction term is written in particle-hole symmetric form so that at half filling ($N = L$) the model is explicitly symmetric under particle-hole transformation when no site-energy offsets are present.

Unless otherwise stated, we set $\hbar = e = 1$ and we characterize dimerization by the dimensionless ratio t_2/t_1. The half-filled ground state is obtained in the total spin-projection sector $S_z = 0$. When singlet-triplet splitting is reported, the triplet energy is computed in the $S_z = 1$ sector (equivalently $2\,S_z = 2$ in the S_z-conserving implementation), and $\Delta_{\text{ST}} = E(S = 1) - E(S = 0)$ is extracted from the difference between converged ground-state energies in the two sectors.

S1.2 DMRG setup and convergence tests

All ground-state calculations in this Supporting Information are performed using the Block2 tensor-network library, employing finite-system DMRG with a matrix-product-state (MPS) representation for the open-boundary Hamiltonian in Eq. S1.[1] We target fixed quantum-number sectors by conserving total particle number N and spin projection S_z(implemented as fixed $N_\uparrow$ and $N_\downarrow$). Unless otherwise stated, calculations are carried out at half filling ($N = L$) in the $S_z = 0$ sector.

DMRG optimization is performed using multiple sweeps with a controlled increase of the maximum bond dimension χ (maxM). Convergence is assessed by monitoring three diagnostics: the ground-state energy E_0, the sweep-to-sweep energy change $|\Delta E| = |E_s - E_{s-1}|$, and the discarded weight. **Fig. S1** shows representative convergence of E_0, $|\Delta E|$, and discarded weight as a function of sweep index for several χ. **Fig. S2** summarizes the final converged E_0 and discarded weight versus χ, demonstrating that the static observables reported in the main text are insensitive to further increases in χ and additional sweeps within our numerical resolution.

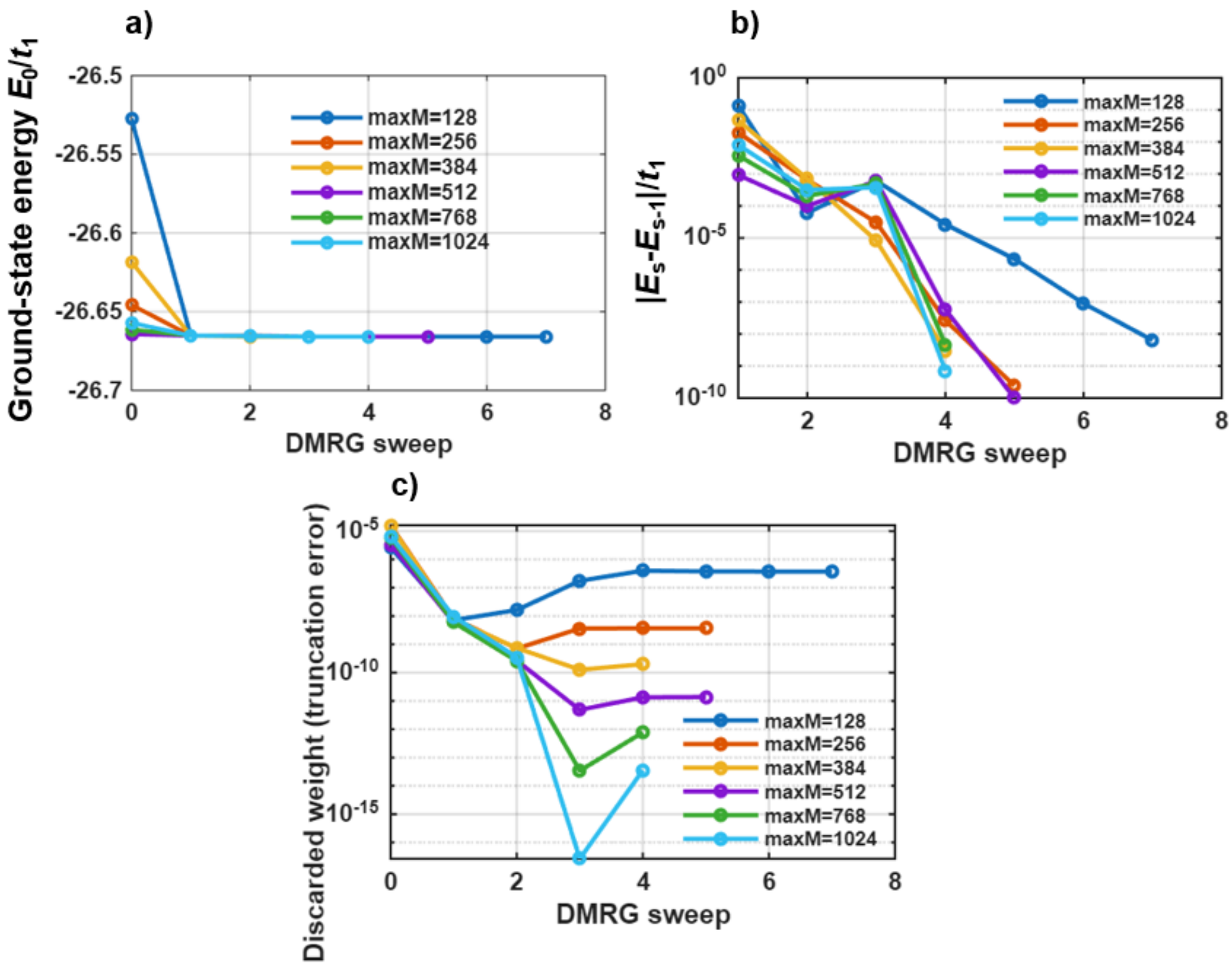

Fig. S1 (a) Ground-state energy E_0/t_1 as a function of DMRG sweep index for the SSH-Hubbard chain at $N = 20$, $t_2/t_1 = 1.5$, and $U/t_1 = 1$, shown for several maximum bond dimensions χ (maxM). (b) Sweep-to-sweep energy change $| E_s - E_{s-1} |/t_1$ on a logarithmic scale. (c) Maximum discarded weight per sweep (truncation error estimate) on a logarithmic scale. Together these panels demonstrate systematic convergence with both sweep number and χ.

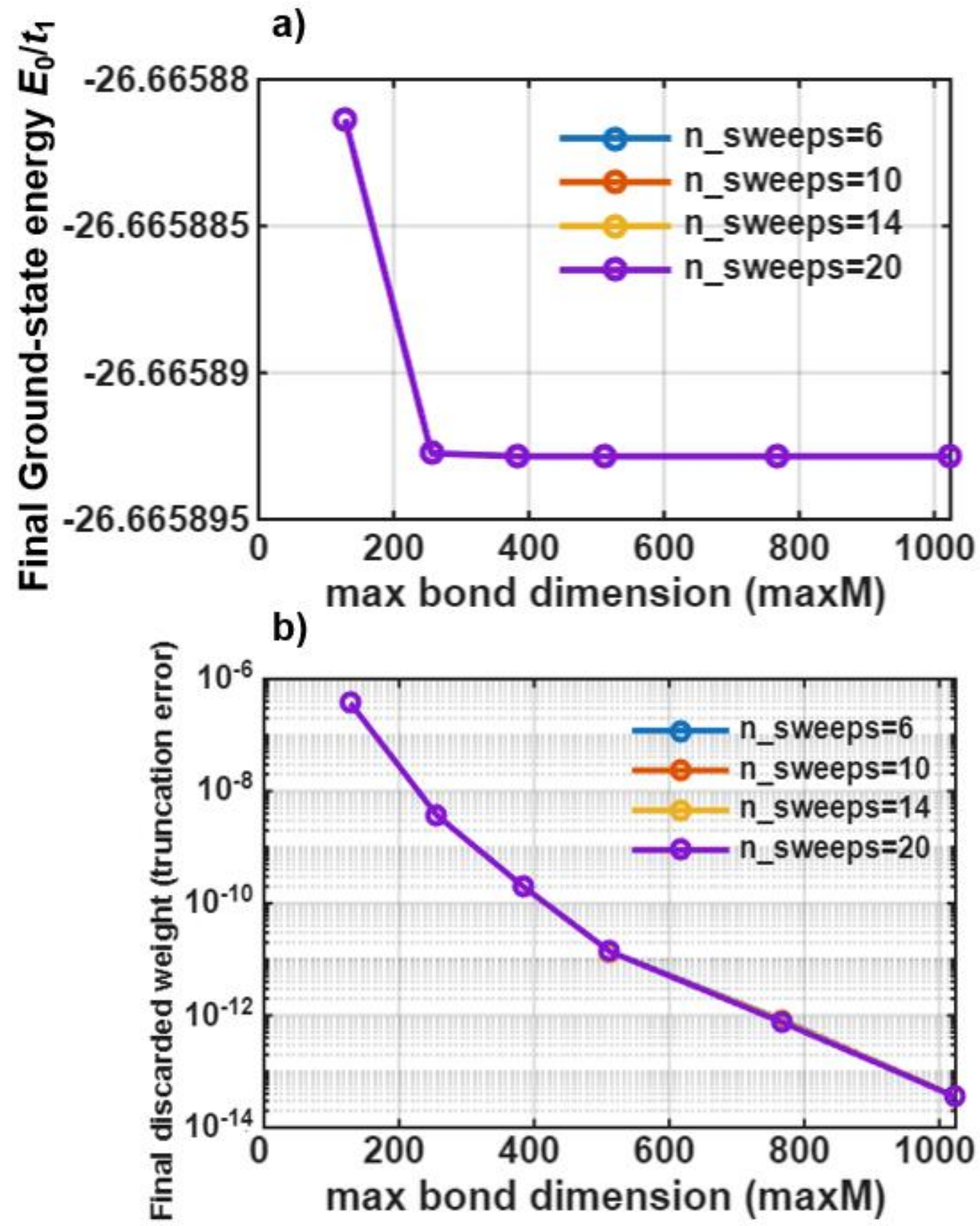

Fig. S2 (a) Final converged ground-state energy E_0/t_1 versus maximum bond dimension χ for $N = 20$, $t_2/t_1 = 1.5$, and $U/t_1 = 1$ (curves correspond to different total sweep counts used in the convergence scan). (b) Final maximum discarded weight versus χ on a logarithmic scale. The saturation of $E_0$with decreasing discarded weight confirms that the ground-state results used in the main text are converged with respect to the MPS bond dimension. As the increase in the number of sweeps above 6 is not affecting the E_0/t_1 and final discarded weight much, they are not visible on the figures.

S1.3 Ground-state observables

All ground-state observables are evaluated on the converged MPS obtained as described in Sec. S1.2. The site-resolved spin density is defined as

$$\langle S_{zi} \rangle = \frac{1}{2} \langle n_{i\uparrow} - n_{i\downarrow} \rangle, \qquad \text{(S2)}$$

and is used to visualize the emergence and spatial localization of edge states in the dimerized chain.

Fig. S3a-c shows $\langle S_{zi} \rangle$ profiles for fixed $U/t_1 = 2$ at three dimerizations $t_2/t_1 = 1.2$, 1.5, and 2 illustrating the progressive localization of spin density toward the chain termini as t_2/t_1 increases.

Fig. S3d provides an additional representative profile at t_2/t_1= 1.5, and U/t_1 = 1, demonstrating that the edge-localization trend persists at more moderate interaction strength.

To quantify the effective end-to-end coupling between terminal moments, we compute the edge-edge spin correlation: (summarized in **Fig. S4**):

$$\langle S_{z1} S_{zL} \rangle \qquad \text{(S3)}$$

This correlation provides an independent ground-state diagnostic of the residual interaction between the terminal moments that underlies the singlet-triplet physics discussed in the main text.

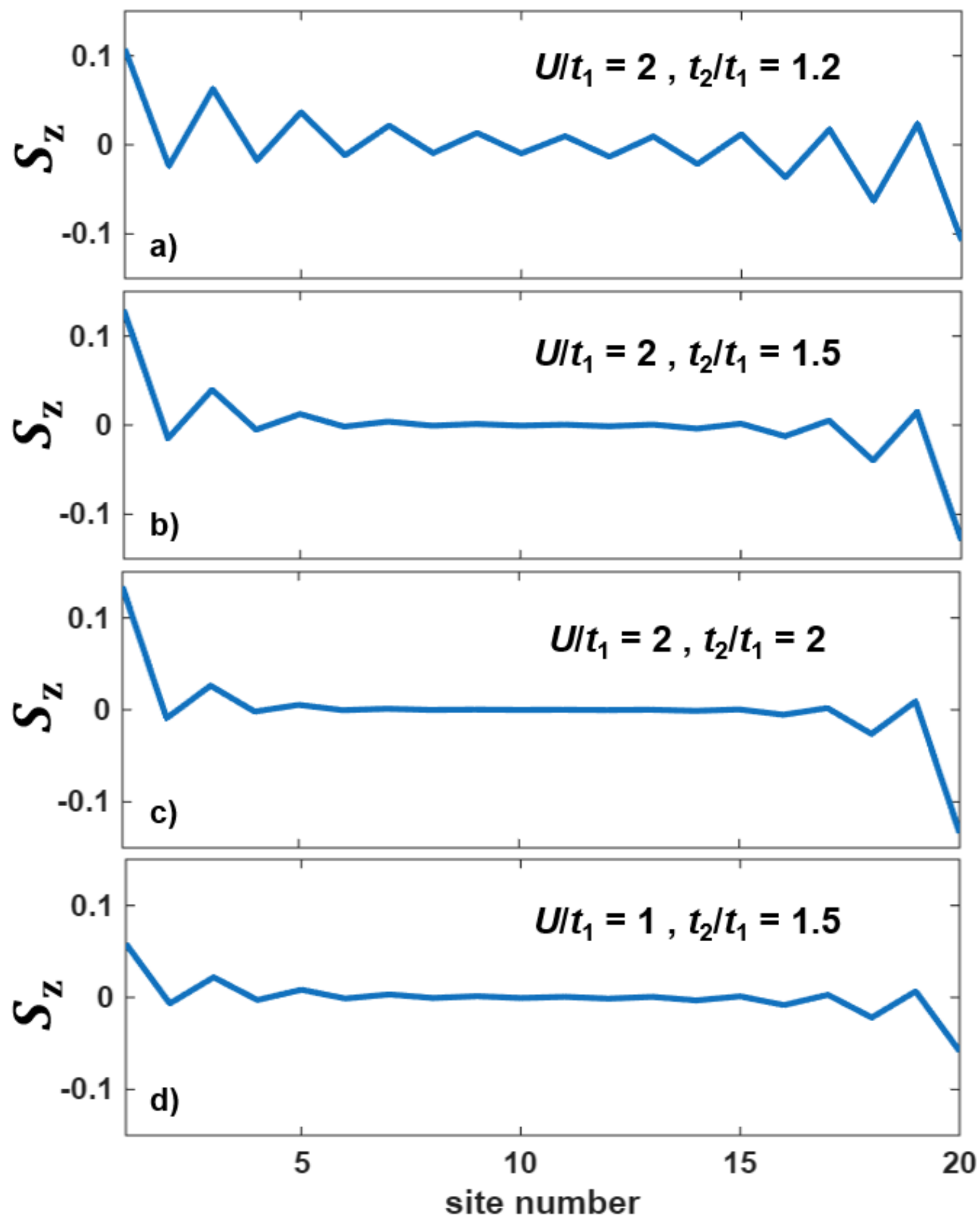

Fig. S3 (a-c) Real-space spin density profiles $\langle S_{zi} \rangle = \frac{1}{2}(\langle n_{i\uparrow} \rangle - \langle n_{i\downarrow} \rangle)$ for an open SSH-Hubbard chain with $U/t_1 = 2$ at three dimerizations: (a) $t_2/t_1 = 1.2$, (b) $t_2/t_1 = 1.5$, and (c) $t_2/t_1 = 2$. Increasing t_2/t_1 concentrates the spin density toward the termini, consistent with progressively

stronger edge localization. (d) Corresponding $\langle S_{zi} \rangle$ profile for $t_2/t_1 = 1.5$, and $U/t_1 = 1$ illustrating that edge-localized spin polarization persists at more moderate interaction strength.

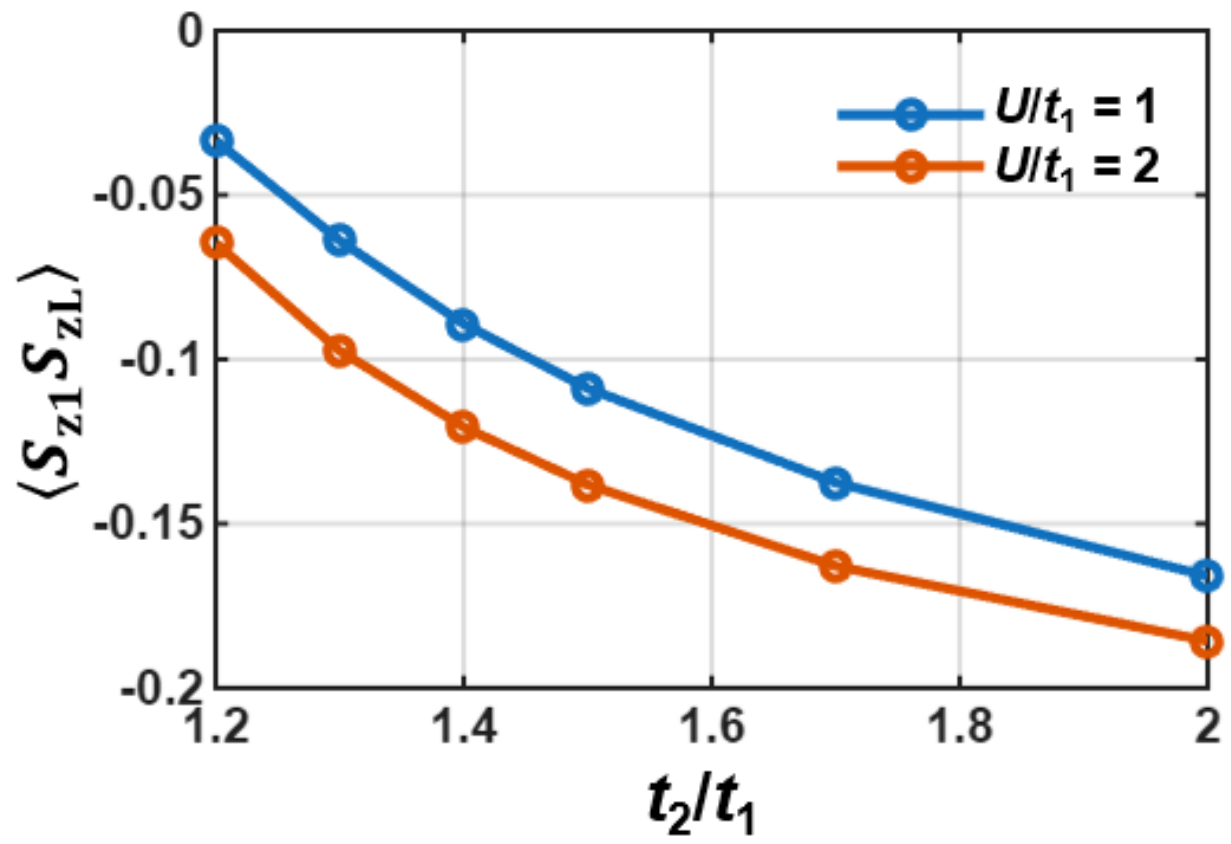

Fig. S4 Edge-edge longitudinal spin correlation $\langle S_{z1} S_{zL} \rangle$ for the open SSH-Hubbard chain as a function of dimerization t_2/t_1, computed in the half-filled $S_z = 0$ ground state and $N = 20$. Data is shown for $U/t_1 = 1$ and $U/t_1 = 2$. The increasingly negative $\langle S_{z1} S_{zN} \rangle$ with larger t_2/t_1 and U/t_1 reflects stronger formation of antiferromagnetically correlated terminal moments. This complements the singlet-triplet gap analysis in the main text, where the energetic splitting decreases as edge-state overlap and virtual charge fluctuations are reduced.

S2. Dynamical DMRG (DDMRG) and Meir-Wingreen (MW) Transport

S2.1 Retarded Green's tensor in correction-vector DDMRG

To embed a fully correlated backbone into a transport junction we require the frequency-resolved, interacting one-particle response of the isolated SSH-Hubbard device. All dynamical calculations in this work are performed using the Block2 tensor-network library, employing dynamical DMRG (DDMRG) in the correction-vector formulation. The central quantity computed by DDMRG is the retarded Green's tensor evaluated with respect to the correlated ground state $H_{\text{dev}} \mid \Psi_0 \rangle = E_0 \mid \Psi_0 \rangle$.[2, 3]

$$G_{0\,ij,\sigma}^{\,r}(\omega) = \langle \Psi_0 \mid c_{i\sigma} \frac{1}{\omega + i\eta + E_0 - H_{\text{dev}}} c_{j\sigma}^{\dagger} \mid \Psi_0 \rangle + \langle \Psi_0 \mid c_{j\sigma}^{\dagger} \frac{1}{\omega + i\eta - E_0 + H_{\text{dev}}} c_{i\sigma} \mid \Psi_0 \rangle$$
(S4)

Here $c_{i\sigma}^{\dagger}(c_{i\sigma})$ creates (annihilates) an electron of spin $\sigma \in \{\uparrow, \downarrow\}$ on site i, and $i, j \in \{1, \ldots, L\}$ label sites of the finite open chain. The two terms in Eq. S4 correspond, respectively, to the particle-addition and particle-removal sectors; together they form the full causal retarded response. The

parameter $\eta > 0$ provides a Lorentzian broadening that sets the spectral resolution and regularizes the resolvent.

In the correction-vector formulation, Eq. S4 is evaluated by solving linear systems for frequency-dependent particle and hole correction vectors,

$$(\omega + i\eta + E_0 - H_{\text{dev}}) \mid \phi_{j\sigma}^{+}(\omega)\rangle = c_{j\sigma}^{\dagger} \mid \Psi_0\rangle, (\omega + i\eta - E_0 + H_{\text{dev}}) \mid \phi_{i\sigma}^{-}(\omega)\rangle = c_{i\sigma} \mid \Psi_0\rangle,$$
(S5)

so that the desired Green's-function element is obtained from simple overlaps,

$$G_{ij,\sigma}^{r}(\omega) = \langle \Psi_0 \mid c_{i\sigma} \mid \phi_{j\sigma}^{+}(\omega)\rangle + \langle \Psi_0 \mid c_{j\sigma}^{\dagger} \mid \phi_{i\sigma}^{-}(\omega)\rangle. \quad \text{(S6)}$$

The advanced Green's function is $G^{a}(\omega) = [G^{r}(\omega)]^{\dagger}$. From G^{r} we also define the interacting spectral tensor,

$$A_{0ij,\sigma}(\omega) = -\frac{1}{\pi} \operatorname{Im} G_{0}{}_{ij,\sigma}^{r}(\omega), \quad \text{(S7)}$$

with local spectra given by $A_{i,\sigma}(\omega) = A_{ii,\sigma}(\omega)$.

The correlated Green's function of the isolated backbone obtained above is then used to construct the steady-state current within the MW and NEGF formalisms. [4, 5] In this setting, interactions are retained through the fully interacting isolated backbone Green's function, while the electrodes enter exactly through energy-dependent lead self-energies; the current is then determined by the non-equilibrium occupations encoded in the lesser Green's function $G^{<}(\omega)$. Because the isolated backbone is evaluated at thermal equilibrium, its lesser Green's function ($G_0^{<}(\omega)$) follows from the fluctuation-dissipation relation,

$$G_0^{<}(\omega) = f(\omega)\,[G_0^{a}(\omega) - G_0^{r}(\omega)] \quad \text{(S8)}$$

with $f(\omega)$ the Fermi function at the device chemical potential and $G_0^{a}(\omega) = [G_0^{r}(\omega)]^{\dagger}$.

The effect of the electrodes and the applied bias is introduced through lead self-energies. The total retarded/advanced and lesser self-energies are written as

$$\Sigma^{r/a}(\omega) = \Sigma_L^{r/a}(\omega) + \Sigma_R^{r/a}(\omega), \Sigma^{<}(\omega) = \Sigma_L^{<}(\omega) + \Sigma_R^{<}(\omega) \quad \text{(S9)}$$

where *L/R* denotes the left/right electrode. The non-equilibrium occupations imposed by the leads enter through the Keldysh components,

$$\Sigma_\alpha^{<}(\omega) = i\, f_\alpha(\omega)\, \Gamma_\alpha(\omega), \qquad f_\alpha(\omega) = f(\omega - \mu_\alpha), \quad \mu_{L/R} = \pm V_{\text{bias}}/2 \quad \text{(S10)}$$

where $\alpha \in \{L, R\}$. To specify these quantities, we model each electrode as a semi-infinite one-dimensional tight-binding chain with nearest-neighbor hopping t_{lead}, coupled to the terminal device sites $\ell_L = 1$ and $\ell_R = N$ through a hopping $t_c^{(\alpha)}$. This yields the retarded self-energy and broadening function

$$\Sigma_\alpha^r(\omega) = |\, t_c^{(\alpha)} \,|^2 \; g_{\text{surf}}^r(\omega) \; |\, \ell_\alpha \rangle\langle \ell_\alpha \,|, \qquad \Gamma_\alpha(\omega) = i[\Sigma_\alpha^r(\omega) - \Sigma_\alpha^a(\omega)] \tag{S11}$$

where the retarded surface Green's function of a semi-infinite tight-binding chain is

$$g_{\text{surf}}^r(\omega) = \begin{cases} \dfrac{\omega - i\sqrt{4t_{\text{lead}}^2 - \omega^2}}{2t_{\text{lead}}^2}, & |\,\omega\,| < 2\,|\,t_{\text{lead}}\,|, \\[2ex] \dfrac{\omega - \text{sgn}(\omega)\sqrt{\omega^2 - 4t_{\text{lead}}^2}}{2t_{\text{lead}}^2}, & |\,\omega\,| > 2\,|\,t_{\text{lead}}\,|. \end{cases} \tag{S12}$$

The correlated molecular backbone is treated explicitly through its interacting Green's function, whereas the electrodes are represented as noninteracting reservoirs. This approximation neglects explicit electrode correlations, image-charge polarization, electrode-induced screening of the effective interaction U, and bias-dependent charge redistribution at the molecule-electrode interface. Accordingly, the U in the present model should be interpreted as an effective junction parameter rather than a gas-phase molecular constant. The contacted Green's functions are then obtained by combining the interacting molecular response with electrode embedding through Dyson's equation,

$$G^r(\omega) = [(G_0^r(\omega))^{-1} - \Sigma^r(\omega)]^{-1}, \; G^a(\omega) = [(G_0^a(\omega))^{-1} - \Sigma^a(\omega)]^{-1} \tag{S13}$$

The lesser Green's function is constructed using the Kadanoff-Baym relation,[6] which accounts for both equilibrium correlations in the isolated backbone and bias-induced occupations from the leads,

$$G^<(\omega) = [\mathbf{I} + G^r(\omega)\Sigma^r(\omega)]\, G_0^<(\omega)\, [\mathbf{I} + \Sigma^a(\omega)G^a(\omega)] \; + \; G^r(\omega)\Sigma^<(\omega)G^a(\omega) \tag{S14}$$

The first term in Eq. S14 contains the lesser Green's function of the isolated interacting backbone and represents the equilibrium occupation and correlated boundary condition of the molecular region before lead embedding. The second term contains the lead lesser self-energies and describes the nonequilibrium occupations injected by the biased electrodes. In the present DDMRG plus MW construction, both terms are retained. The first keeps the correlated molecular occupation consistent with the DDMRG Green's function, while the second introduces the reservoir driven nonequilibrium population that gives rise to current.

With $G^{r/a/<}(\omega)$ in hand, the steady-state current is evaluated using the MW expression,

$$I(V) = \frac{e}{\hbar}\int \frac{d\omega}{2\pi} \operatorname{Tr}[\Sigma_L^{<}(\omega)\, G^{>}(\omega) - \Sigma_L^{>}(\omega)\, G^{<}(\omega)] \tag{S15}$$

which describes the net electron flow between the left electrode and the interacting device. In the noninteracting limit $U/t_1 = 0$, the MW construction reduces to the corresponding Landauer description, while at finite U/t_1 a self-consistent mean-field Landauer treatment provides a useful static single-particle reference that can be compared with the correlated DDMRG+MW result (**Fig. S9).**

To validate the hybrid frequency-domain transport construction, in the next section we benchmark it against the standard Hubbard dimer.[7] In this minimal correlated system, the interplay between interaction-induced spectral splitting and transport can be characterized independently using established NEGF approaches. We find that the stationary, energy-resolved currents obtained from our DDMRG+MW scheme reproduce the key qualitative features reported in Hartree-Fock and second-Born treatments, including the interaction-driven modification of transport channels (**Fig. S5**). This agreement indicates that the present approach consistently combines the correlated spectral information of the isolated backbone with electrode embedding, capturing the expected signatures of correlated transport in a well-controlled test case.

S2.2 Hubbard dimer benchmark

As a benchmark for our frequency-domain transport workflow, we compare it against the standard Hubbard dimer, a previously used correlated test problem.[7] In the reference study, the two-site spinful Hubbard model is treated within an open-system nonequilibrium Green's-function framework based on Hartree-Fock (HF), second-Born approximation (2BA) and Landauer-Büttiker (LB) treatment. The dimer Hamiltonian uses nearest-neighbor hopping $h = 1$, onsite repulsion U, and chemical potential $\mu = U/2$, so that the system is at half filling. The authors probe the dimer by coupling it into a structured environment composed of many narrow-band left subleads and one broad right lead, and they analyze energy-resolved current under a bias protocol applied to the right lead.

Our approach follows the same hybrid strategy used throughout the main text: we first compute the fully interacting retarded Green's function of the isolated correlated device using correction-vector DDMRG and then embed that Green's function into external electrodes within the MW non-equilibrium Green's function framework to obtain the contacted steady-state current. In our work, this lead embedding is performed through retarded and lesser self-energies, Dyson's equation for the contacted retarded Green's function, the long Kadanoff-Baym construction for the lesser component, and finally the MW current expression.

To match the benchmark as closely as possible, we use the same central correlated model and the same structured lead geometry as in the reference paper.[2] Specifically, the left lead is represented as a collection of narrow Lorentzian subleads with center energies $\epsilon_{1,j}$, widths $\Omega_{1,j}$, and coupling

matrix $\gamma_{1,j}$, while the right lead is represented by a broad Lorentzian centered at $\epsilon_2 = 0$ with width Ω_2 and coupling matrix γ_2. We then compute the channel-resolved steady-state current through each left sublead and plot it as a function of $\epsilon_{1,j}$, which is directly comparable to the reference study. This comparison is therefore intended as a benchmark of the DDMRG plus MW route for correlated transport.

For the weakly correlated case the reference paper uses $U = 1$, $h = 1$, $\mu = U/2$, inverse temperature $\beta = 10$, 21 left subleads with $\Omega_{1,j} = 0.5$ and $\epsilon_{1,j} = -5 + 0.5(j-1)$ for $1 \leq j \leq 21$, and a right lead with $\epsilon_2 = 0$ and $\Omega_2 = 10$. The site-basis tunneling matrices are $\gamma_{1,j} = 0.05\begin{pmatrix}1 & 0\\0 & 0\end{pmatrix}$ and $\gamma_2 = 0.07\begin{pmatrix}0 & 0\\0 & 1\end{pmatrix}$. The two stationary bias states are $V_2 = -2$ and $V_2 = +2$.

For the moderately correlated case, the reference paper changes the lead resolution to better resolve the spectral features at larger interaction. In that case, $U = 5$, $\mu = U/2$, the right-lead width remains $\Omega_2 = 10$, the left sublead width is reduced to $\Omega_{1,j} = 0.1$, and the number of left subleads is increased to 28 with positions $\epsilon_{1,j} = -2.7 + 0.2(j-1)$. The tunneling matrices are kept at $\gamma_{1,j} = 0.05\begin{pmatrix}1 & 0\\0 & 0\end{pmatrix}$ and $\gamma_2 = 0.07\begin{pmatrix}0 & 0\\0 & 1\end{pmatrix}$, and again the comparison is made between the stationary currents at $V_2 = -2$ and $V_2 = +2$.

As shown in **Fig. S5**, the $U = 1$ results closely reproduce the published benchmark, while for the strong interaction $U = 5$ results the small deviation in peak position relative to the published benchmark is not unexpected, since the reference results are based on approximate HF, 2BA, LB treatments, whereas our calculation uses a nonperturbative interacting Green's function; we therefore view this comparison as a validation of the method and the overall trends rather than as a point-by-point quantitative reproduction of the approximate reference calculations.

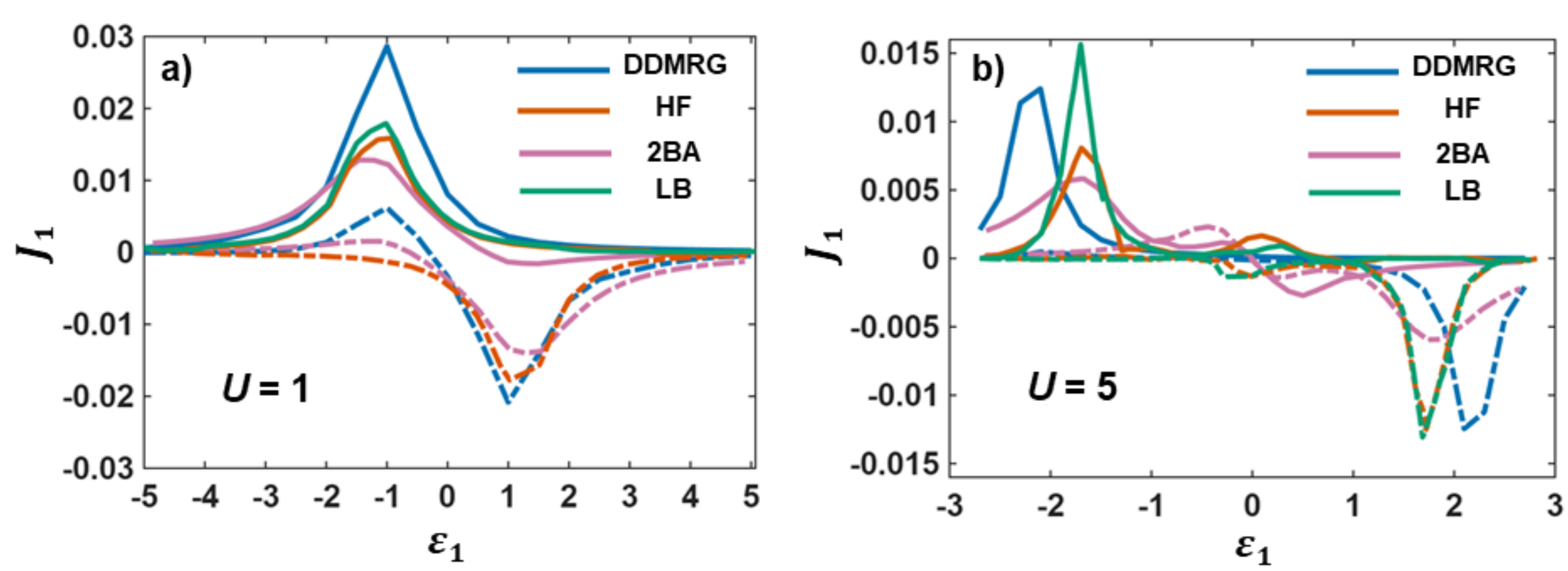

Fig. S5. Channel-resolved steady-state current through the left probe lead, J_1, plotted as a function of probe energy ϵ_1 for the half-filled Hubbard dimer under the two stationary right-lead biases $V_2 = -2$ (solid lines) and $V_2 = +2$ (dashed lines). (a) $U = 1$. (b) $U = 5$. In both panels, the interacting retarded Green's function of the isolated dimer was computed with dynamical density

matrix renormalization group and embedded into structured electrodes within the MW nonequilibrium Green's function framework.

S2.3 Broadening dependence of the MW integrand (effective transmission).

In correction-vector DDMRG the retarded Green's tensor $G^r(\omega)$ is evaluated at discrete frequencies ω_k with a finite imaginary part $\eta > 0$ in the resolvent. The parameter η provides a controlled Lorentzian broadening that regularizes the linear solves and sets the effective spectral resolution. In practice, decreasing η narrows spectral features without changing their physical locations, provided the underlying calculation is converged and $\Delta\omega$ is sufficiently small.

To verify that the transport-relevant spectral structure is not an artifact of the chosen broadening, **Fig. S6** compares the effective transmission (MW integrand) $\mathcal{T}_{\mathrm{MW}}(\omega)$ for four broadenings, $\eta/t_1 = 2 \times 10^{-3}$, 1×10^{-3}, 5×10^{-4}, and 1×10^{-4}. As expected, reducing η slightly sharpens the resonances, but the peak positions and overall low-energy weight distribution remain unchanged within numerical resolution. This confirms that the MW integrands are robust with respect to the broadening used in the DDMRG evaluation.

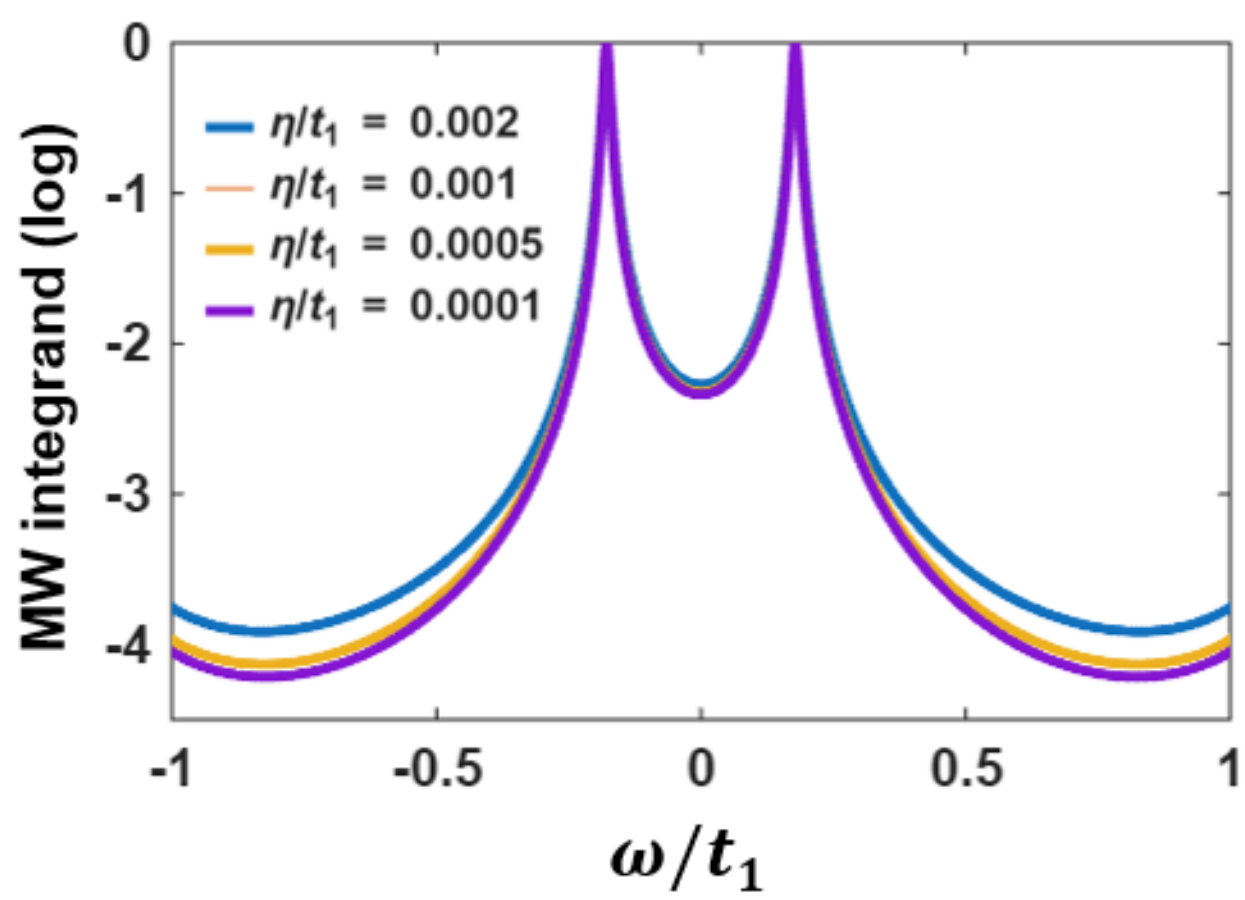

Fig. S6 The MW current integrand spectra for an $N = 8$ SSH segment with $t_2/t_1 = 1.5$, and $U/t_1 = 0$ shown for four Lorentzian broadenings used in the DDMRG evaluation of the Green's function: $\eta/t_1 = 2 \times 10^{-3}$, 1×10^{-3}, 5×10^{-4}, and 1×10^{-4} (in units of t_1). Decreasing η/t_1 slightly sharpens the resonance features as expected while leaving their positions and overall spectral-weight distribution unchanged, confirming that the effective transmission entering the current integration is robust with respect to the chosen broadening.

S2.4 Trivial *vs* topological regime control calculations for the SSH-Hubbard chain

To provide a control for the edge-state-mediated transport mechanism discussed in the main text, we also examined the topologically trivial dimerization regime of the SSH-Hubbard chain. For the open-chain termination used here, $t_2/t_1 < 1$ corresponds to the trivial regime, whereas $t_2/t_1 > 1$

supports end-derived states. This comparison allows us to separate transport associated with topological end states from generic effects of dimerization and electron-electron interaction.

Fig. S7a shows the DDMRG+MW transmission map as a function of energy and dimerization ratio at fixed interaction strength. **Fig. S7b** depicts the length dependence of the transmission in the trivial regime for $t_2/t_1 = 0.8$ and $U/t_1 = 1.5$. In contrast to the weak length dependence observed in the topological regime, the low-bias transmission decreases with increasing chain length. These results confirm that the weak length dependence emphasized in the main text is not a generic consequence of dimerization or interaction alone but requires the end-derived states present in the $t_2/t_1 > 1$ regime.

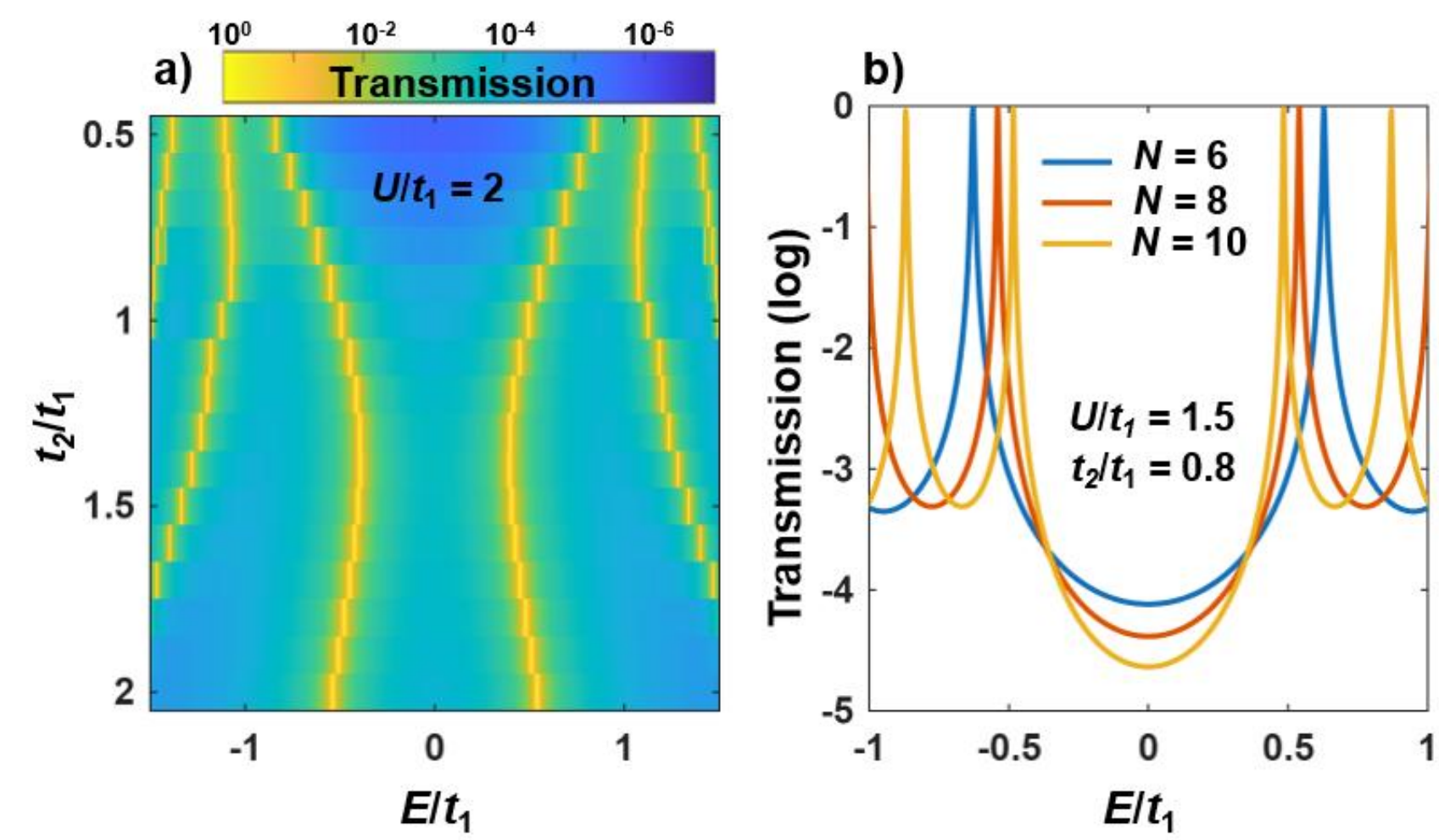

Fig. S7. Trivial-regime control calculations for the SSH-Hubbard chain. (a) DDMRG + MW transmission map as a function of energy E/t_1 and dimerization ratio t_2/t_1 at fixed $U/t_1 = 2$. The scan spans both the topologically trivial regime, $t_2/t_1 < 1$, and the topological regime, $t_2/t_1 > 1$. (b) Length dependence of the transmission in the trivial regime for $t_2/t_1 = 0.8$ and $U/t_1 = 1.5$, shown for $N = 6, 8,$ and 10. The low-bias transmission decreases with increasing chain length. These control calculations show that weak length dependence is not a generic consequence of dimerization or interaction alone but requires the edge-derived states present in the $t_2/t_1 > 1$ regime.

S2.5 Introducing a staggered donor-acceptor onsite potential

To test the effect of one-electron asymmetry on the transport mechanism, we introduce a staggered donor-acceptor onsite potential to the SSH-Hubbard backbone,

$$H_{\mathrm{DA}} = \sum_{i,\sigma} \epsilon_i \, n_{i\sigma}, \qquad \epsilon_i = \delta_{\mathrm{DA}}(-1)^i \tag{S16}$$

This onsite-potential perturbation is a chemically meaningful representation of donor-acceptor one-electron asymmetry, because chemically distinct repeat units or alternating electron-rich and electron-poor sites naturally produce nonuniform local orbital energies along the backbone.

For even chains, this perturbation has zero spatial average and therefore does not simply shift the entire molecular spectrum with respect to the Fermi level. Instead, it alternates the onsite energies along the backbone and mimics a donor-acceptor imbalance between neighboring sites. This term breaks the spatial symmetry of the wavefunction and tests how sensitive the transport channels are to one-electron site-energy asymmetry.

Breaking this symmetry does not necessarily remove edge-localized states immediately. However, it changes their energetic alignment and can reduce the balance of their coupling to the two electrodes. **Fig. S8a** shows the DDMRG+MW transmission map for $U/t_1 = 0$, $N = 8$, and $t_2/t_1 = 1.5$ as a function of δ_{DA}/t_1. In the absence of interaction, increasing the staggered onsite potential shifts and redistributes the low-energy resonances. Importantly, the resulting donor-acceptor asymmetry reduces the left-right balance of the transport-active states, so that a resonance can couple more strongly to one electrode than to the other. Since a resonant transmission peak reaches unity only for symmetric coupling to the two electrodes, this imbalance suppresses the low-bias conductance. This behavior indicates that one-electron donor-acceptor asymmetry moves the system away from the ideal balanced edge-state transport regime and toward more conventional off-resonant length-dependent transport. The corresponding length dependence at $\delta_{\mathrm{DA}}/t_1 = 0.4$, shown in **Fig. S8b**, confirms this trend. For $U/t_1 = 0$, the low-bias transmission decreases with increasing chain length, consistent with closed-shell off-resonant transport.

The interacting case behaves remarkably differently from the noninteracting case. **Fig. S8c** shows the corresponding transmission map for $U/t_1 = 1.5$. At moderate δ_{DA}/t_1, the interaction partially counteracts the one-electron asymmetry by stabilizing open-shell character and maintaining low-energy resonant channels. As a result, the resonances first move closer together before separating again at larger δ_{DA}/t_1, reflecting competition between donor-acceptor onsite imbalance and correlation-driven open-shell behavior. The length dependence at $U/t_1 = 1.5$ and $\delta_{\mathrm{DA}}/t_1 = 0.4$, shown in **Fig. S8d**, demonstrates that the transmission remains strong over the finite chain lengths studied. This behavior contrasts with the noninteracting case and shows that moderate electron-electron interaction can preserve transport-active correlated channels even in the presence of one-electron donor-acceptor asymmetry.

Overall, a staggered donor-acceptor potential shifts and redistributes the low-energy resonances, and sufficiently strong asymmetry suppresses low-bias transport. However, at moderate asymmetry and finite interaction, edge-derived transport channels can remain active, supporting the conclusion that long-range transport is governed by the balance between dimerization, interaction strength, and many-body level alignment.

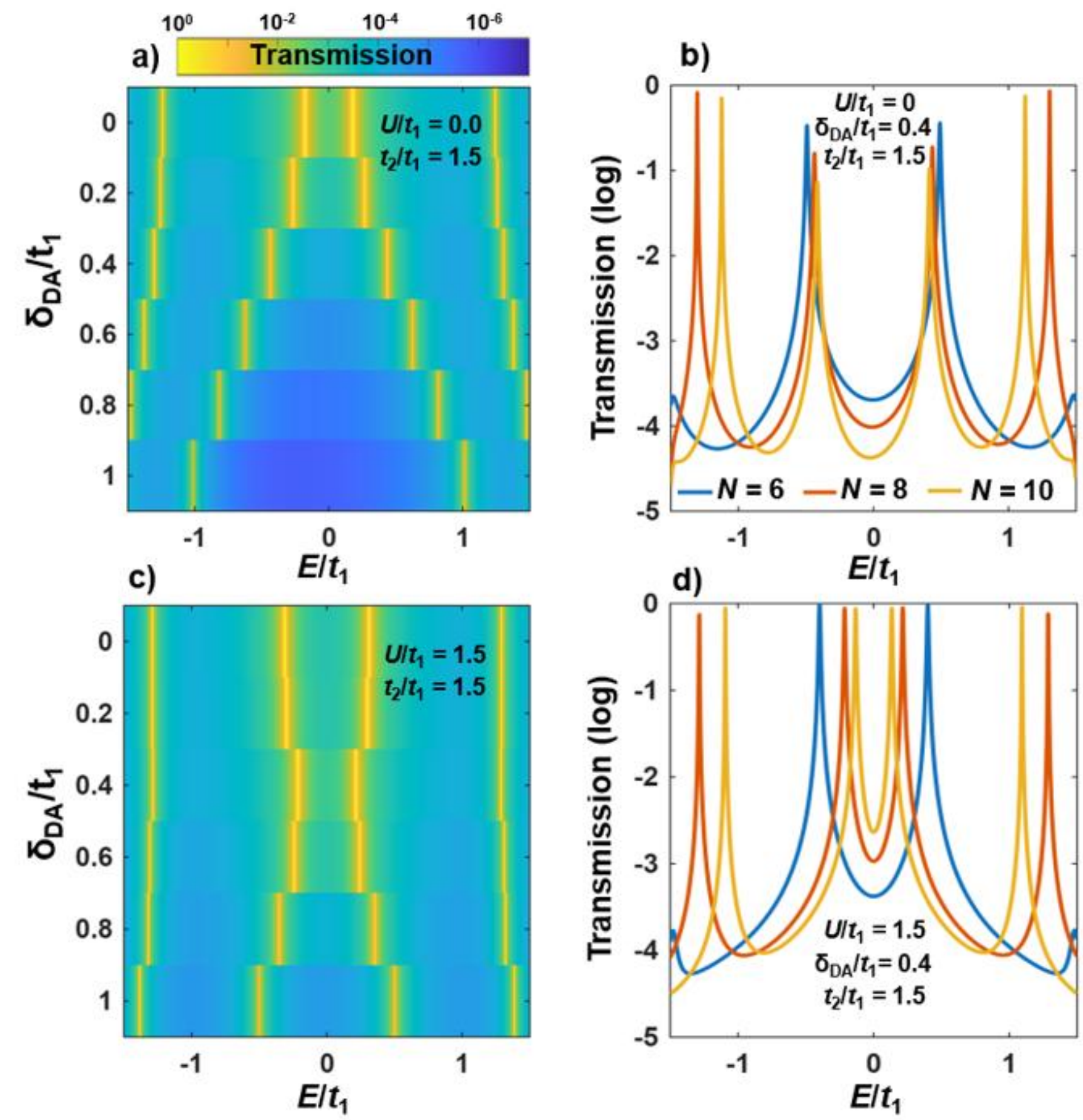

Fig. S8. DDMRG/MW transmission spectra for an SSH-Hubbard chain with $t_2/t_1 = 1.5$ after introducing a staggered donor-acceptor onsite potential. (a) Transmission map versus E/t_1 and δ_{DA}/t_1 for $N = 8$ and $U/t_1 = 0$. (b) Length dependence at $U/t_1 = 0$ and $\delta_{\mathrm{DA}}/t_1 = 0.4$ for $N = 6, 8, 10$, showing length-dependent suppression of low-bias transmission. (c) Corresponding transmission map for $N = 8$ and $U/t_1 = 1.5$. (d) Length dependence at $U/t_1 = 1.5$ and $\delta_{\mathrm{DA}}/t_1 = 0.4$, where transmission remains strong and increases over the finite lengths studied.

S2.6 Mean-field Landauer reference calculations

To clarify the relation between the correlated DDMRG+MW calculation and a conventional single-particle transport treatment, we performed additional mean field Landauer reference calculations for the same SSH-Hubbard junction. In the noninteracting limit $U/t_1 = 0$, the SSH-Hubbard Hamiltonian reduces to a single-particle tight-binding model. In this limit, the DDMRG Green's function reduces to the corresponding single-particle Green's function, and the MW current gives the same transmission as the Landauer formula within the numerical broadening used in the calculation.

For finite U/t_1, the mean field Landauer calculation is not an exact interacting transport calculation. Instead, it is a static single-particle approximation to the interacting problem. The Hubbard interaction is decoupled self-consistently as

$$U\left(n_{i\uparrow}-\frac{1}{2}\right)\left(n_{i\downarrow}-\frac{1}{2}\right)\rightarrow$$

$$U(n_{i\uparrow}-1/2)(\langle n_{i\downarrow}\rangle-1/2)+U(\langle n_{i\uparrow}\rangle-1/2)(n_{i\downarrow}-1/2)-U(\langle n_{i\uparrow}\rangle-1/2)(\langle n_{i\downarrow}\rangle-1/2).$$

(S17)

This decoupling produces a self-consistent spin-dependent one-particle Hamiltonian. Once the interaction has been absorbed into this static effective potential, the resulting problem is single-particle in form, and its transport can be evaluated using the Landauer formula. Thus, the finite-U mean field Landauer result should be viewed as a comparison to a commonly used static single-particle approximation rather than as an exact interacting transport result.

This comparison highlights the difference between static mean field transport and the correlated DDMRG + MW treatment. The mean field approach captures interaction effects only through spin-dependent onsite potentials, whereas DDMRG resolves the correlated many-body addition and removal excitations directly. Therefore, deviations between the two approaches at finite U/t_1 indicate where the static single-particle picture begins to depart from the correlated many-body transport description.

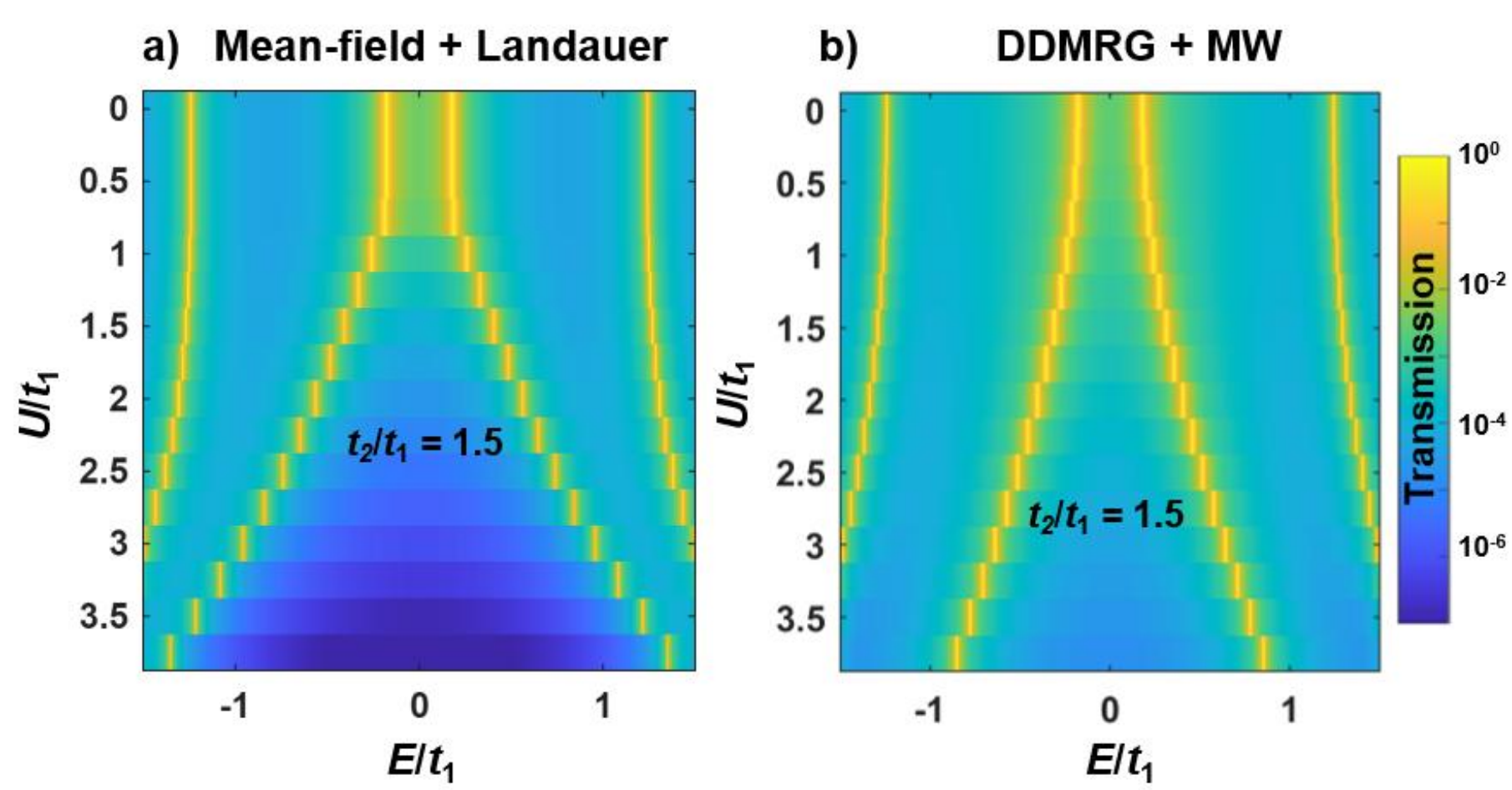

Fig. S9. Comparison of correlated and mean-field transmission as a function of interaction strength. Transmission maps for an $N = 8$ SSH-Hubbard chain with $t_2/t_1 = 1.5$, plotted as a function of energy and interaction strength U/t_1. (a) Mean-field Landauer transmission. (b) Correlated DDMRG+MW transmission obtained using the same SSH-Hubbard parameters. At $U/t_1 = 0$, the correlated calculation reduces to the non-interacting Landauer limit, and the two approaches show the same resonance structure. As U/t_1 increases, the resonance positions begin to deviate, reflecting the difference between correlated electron-addition/removal excitations captured by DDMRG and the static effective-potential description used in mean-field theory.

S3. Time-Dependent DMRG Under Finite Bias

S3.1 Junction geometry for time evolution (finite leads, couplings)

Real-time transport calculations are performed on a finite lead-device-lead junction in which the interacting SSH-Hubbard segment is contacted by two noninteracting tight-binding leads of finite length. The total time-evolution Hamiltonian is written as:

$$H_{\mathrm{tot}}(t) = H_{\mathrm{device}} + H_L + H_R + H_{\mathrm{couple}} \tag{S18}$$

where H_{dev} is the SSH-Hubbard device Hamiltonian in Eq. S1. Each finite lead is modeled as a spin-degenerate nearest-neighbor chain,

$$H_\alpha = -t_{\mathrm{lead}} \sum_{m=1}^{N_{\mathrm{lead}}-1} \sum_{\sigma} \left(c_{\alpha m\sigma}^{\dagger} c_{\alpha(m+1)\sigma} + \mathrm{h.c.} \right), \alpha \in \{L, R\} \tag{S19}$$

with hopping t_{lead} and N_{lead} sites per lead. The leads are coupled to the terminal sites of the device by a single hopping amplitude,

$$H_{\mathrm{couple}} = -\sum_{\sigma} \left[t_{cL} c_{L1\sigma}^{\dagger} c_{1\sigma} + t_{cR} c_{R1\sigma}^{\dagger} c_{N\sigma} + \mathrm{h.c.} \right] \tag{S20}$$

so that only the device ends are directly contacted. Unless otherwise stated we use symmetric couplings $t_{cL} = t_{cR} = t_{\mathrm{c}}$ and identical left/right leads.

This finite-geometry setup enables explicit time evolution of the full interacting junction. The lead length N_{lead} is chosen large enough that a quasi-steady current plateau develops before boundary reflections return to the device.

S3.2 Bias protocol and quench mitigation

To drive the finite junction out of equilibrium we apply a time-dependent bias that shifts the onsite energies of the left and right leads in opposite directions. Starting from the many-body ground state of the unbiased Hamiltonian ($V = 0$), we switch on a bias V using a smooth hyperbolic-tangent ramp,

$$H_{\mathrm{bias}}(t) = \sum_{\sigma} [\sum_{m\in L} \frac{V(t)}{2} n_{Lm\sigma} - \sum_{m\in R} \frac{V(t)}{2} n_{Rm\sigma}], \qquad V(t) = \frac{V_0}{2}\left[1 + \tanh\left(\frac{t-t_0}{\tau}\right)\right], \tag{S21}$$

where $n_{\alpha m\sigma} = c_{\alpha m\sigma}^{\dagger} c_{\alpha m\sigma}$. The parameter $\tau = 5$ sets the switching timescale (adiabaticity), and $t_0 = 2$ fixes the center of the ramp, for $t \ll t_0$, $V(t) \approx 0$, while for $t \gg t_0$, $V(t) \to V$. The bias is

applied only in the leads (not on the interacting device), so that the transport response arises from charge transfer between reservoirs through the correlated segment.

A smooth ramp is essential to mitigate unphysical transients associated with a sudden quench. In particular, the *tanh* protocol suppresses high-frequency excitations and reduces spurious oscillations in the bond currents, allowing a clear quasi-steady plateau to emerge over a time window set by the lead length. The steady current reported in the main text is obtained by averaging over this plateau after the initial charging transient has decayed and before reflections from the finite lead boundaries reach the device region.

S3.3 Time-evolution parameters (Δt, cutoff, and χ_{evo})

Real-time transport is simulated with the time-dependent DMRG implementation in Block2 using the time dependent variational principle (TDVP)-based time-evolution engine. [1] The many-body state is propagated under the time-dependent Hamiltonian $H(t) = H_{\text{tot}} + H_{\text{bias}}(t)$ in discrete steps. Formally, over one step we apply the short-time propagator evaluated at the midpoint of the interval,

$$\mid \Psi(t+\delta t)\rangle \approx \exp[-\frac{i}{\hbar} H\left(t+\frac{\delta t}{2}\right)\delta t] \mid \Psi(t)\rangle, \qquad \text{(S22)}$$

where δt is the real-time step. In Block2 implementation this evolution is carried out as a sequence of local TDVP sweeps, and the number of sweeps per step is set by the sub-sweep parameter of the time-evolution driver.

The accuracy and stability of the evolution are governed by three numerical controls. First, the timestep δt sets the discretization error of the local propagator. In practice, reducing δt suppresses step-size artifacts in the transient dynamics and improves the quantitative stability of the plateau current. Second, compression of the MPS after local updates is controlled by the truncation threshold (cutoff) used in the singular-value truncations. Block2 reports the associated discarded weight during the run, which provides a direct measure of the information removed by truncation. Third, the maximum bond dimension allowed during time evolution, denoted here by χ_{evo}, is set through the evolution bond-dimension schedule. Increasing χ_{evo}allows the MPS to accommodate the entanglement growth induced by the bias quench and typically extends the time window over which a clear quasi-steady current plateau can be resolved before truncation errors accumulate.

In addition, the TDVP propagation in Block2 evaluates local matrix exponentials using a Krylov-based procedure; its numerical accuracy is controlled by the Krylov convergence threshold and the maximum Krylov subspace size. In the calculations reported in the main text these parameters are tightened as needed so that the extracted steady current is insensitive to further refinement of the local propagation accuracy.

Convergence of the reported tDMRG currents is established by verifying that the plateau-averaged current remains unchanged within numerical resolution upon (i) reducing Δt, (ii) tightening the

truncation cutoff, and (iii) increasing χ_{evo}, while simultaneously ensuring that the discarded weight remains small and the MPS norm stays close to unity over the plateau window.

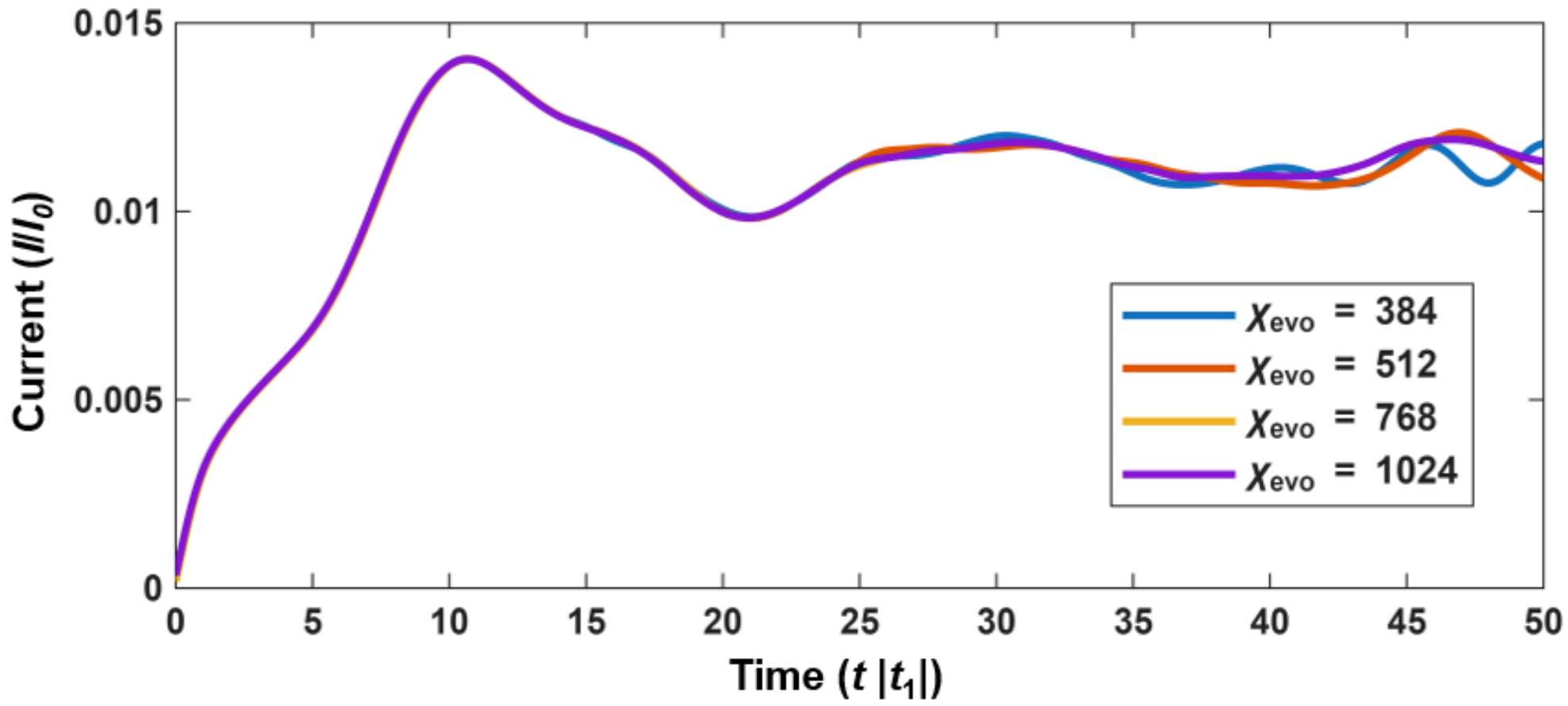

Fig. S10. Convergence of the time-dependent current with the evolution bond dimension in tDMRG/TDVP. The current $I(t)$ is shown for evolution bond dimensions $\chi_{\text{evo}} = 384, 512, 768$, and 1024 while keeping all other parameters fixed: finite leads $t_{\text{lead}}/t_1 = 1$, lead-device coupling $t_c/t_1 = 0.1$; SSH-Hubbard device with $t_2/t_1 = 1.5$ and $U/t_1 = 1$. Bias ramp $V(t) = V_0[1 + tanh((t - t_0)/\tau)]/2$ with $V_0/t_1 = 1.2$, $t_0|t_1| = 2$, and $\tau|t_1| = 5$. Time step $\Delta t|t_1| = 0.05$ and total propagation time $T_{\text{total}}|t_1| = 50$. The collapse of the $I(t)$ traces as χ_{evo} increases demonstrates that the transient response and the quasi-steady current plateau are converged with respect to entanglement growth during the evolution. Current is reported in units of $I_0 = et_1/\hbar$. In our numerical units, $e = \hbar = 1$

The time-resolved site occupations and spin densities provide a dynamical picture consistent with the static DMRG and DDMRG analyses (**Fig. S11**). For $U/t_1 > 0$ and $t_2/t_1 > 1$, the main transport channels are edge states that are already visible in the ground-state DMRG profiles. After the bias ramp, the spin-resolved occupations on the terminal sites show only small transient deviations from their initial values, and these relax as the current approaches its quasi-steady plateau. The absence of long-lived occupation oscillations indicates efficient coupling of the edge states to the electrodes, so carriers injected at the termini are drained into the lead continua rather than remaining trapped in repeated interfacial reflections.

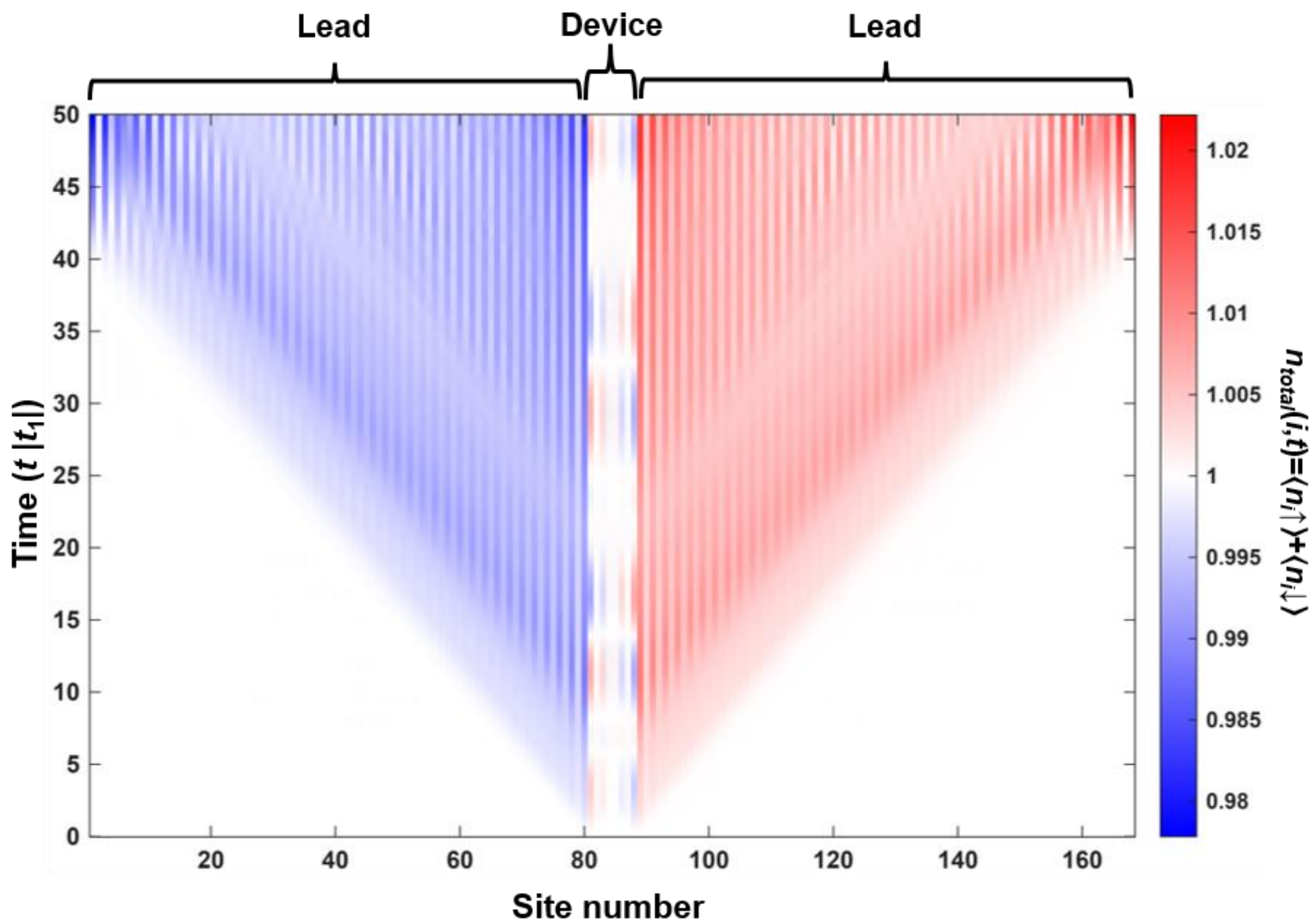

Fig. S11. Time- and site-resolved total occupation during the real-time transport simulation. The color map shows $n_{\mathrm{tot}}(i,t) = \langle n_{i\uparrow}(t)\rangle + \langle n_{i\downarrow}(t)\rangle$ as a function of lattice site i and time t, obtained from the TDVP time evolution after switching on the bias $V(t)$. The setup parameters are the same as in **Fig. S10**.

References

(1) Zhai, H.; Larsson, H. R.; Lee, S.; Cui, Z.-H.; Zhu, T.; Sun, C.; Peng, L.; Peng, R.; Liao, K.; Tölle, J.; et al. Block2: a comprehensive open source framework to develop and apply state-of-the-art DMRG algorithms in electronic structure and beyond. *J. Chem. Phys.* **2023**, *159*, 234801, DOI: 10.1063/5.0180424.

(2) Ronca, E.; Li, Z.; Jimenez-Hoyos, C. A.; Chan, G. K.-L. Time-step targeting time-dependent and dynamical density matrix renormalization group algorithms with ab initio Hamiltonians. *J. Chem. Theory Comput.* **2017**, *13*, 5560–5571, DOI: 10.1021/acs.jctc.7b00682.

(3) Jeckelmann, E. Dynamical density-matrix renormalization-group method. *Phys. Rev. B* **2002**, *66*, 045114, DOI: 10.1103/PhysRevB.66.045114.

(4) Jauho, A.-P.; Wingreen, N. S.; Meir, Y. Time-dependent transport in interacting and noninteracting resonant-tunneling systems. *Phys. Rev. B* **1994**, *50*, 5528–5544, DOI: 10.1103/PhysRevB.50.5528.

(5) Meir, Y.; Wingreen, N. S. Landauer formula for the current through an interacting electron region. *Phys. Rev. Lett.* **1992**, *68*, 2512–2515, DOI: 10.1103/PhysRevLett.68.2512.

(6) Haug, H.; Jauho, A.-P. *Quantum Kinetics in Transport and Optics of Semiconductors*; Solid-State Sciences; Springer, **2008**, DOI: 10.1007/978-3-540-73564-9.

(7) Pavlyukh, Y.; Tuovinen, R. Nonequilibrium transport through the Hubbard dimer. *Eur. Phys. J. Spec. Top.* **2025**, DOI: 10.1140/epjs/s11734-025-01768-9.
